# Enhanced RNA knockdown efficiency with engineered fusion guide RNAs that function with both CRISPR-CasRx and hammerhead ribozyme

**DOI:** 10.1186/s13059-023-02852-w

**Published:** 2023-01-17

**Authors:** Yonghao Zhan, Congcong Cao, Aolin Li, Hongbing Mei, Yuchen Liu

**Affiliations:** 1 Shenzhen Institute of Translational Medicine, Shenzhen Second People’s Hospital, The First Affiliated Hospital of Shenzhen University, Health Science Center, Shenzhen University, Shenzhen, 518035 China; 2 Department of Urology, The First Affiliated Hospital of Zhengzhou University, No. 1 Jianshe East Road, Zhengzhou, 450052 China; 3 Shenzhen Institute of Synthetic Biology, Shenzhen Institutes of Advanced Technology, Chinese Academy of Sciences, Shenzhen, 518055 China; 4 Department of Urology, Shenzhen Second People’s Hospital, The First Affiliated Hospital of Shenzhen University, International Cancer Center, Shenzhen University School of Medicine, Shenzhen, 518035 China

## Abstract

**Background:**

CRISPR-Cas13 is a newly emerging RNA knockdown technology that is comparable to RNAi. Among all members of Cas13, CasRx degrades RNA in human cells with high precision and effectiveness. However, it remains unclear whether the efficiency of this technology can be further improved and applied to gene therapy.

**Results:**

In this study, we fuse CasRx crRNA with an antisense ribozyme to construct a synthetic fusion guide RNA that can interact with both CasRx protein and ribozyme and tested the ability of this approach in RNA knockdown and cancer gene therapy. We show that the CasRx-crRNA-ribozyme system (CCRS) is more efficient for RNA knockdown of mRNAs and non-coding RNAs than conventional methods, including CasRx, shRNA, and ribozyme. In particular, CCRS is more effective than wild-type CasRx when targeting multiple transcripts simultaneously. We next use bladder cancer as a model to evaluate the anticancer effects of CCRS targeting multiple genes in vitro and in vivo. CCRS shows a higher anticancer effect than conventional methods, consistent with the gene knockdown results.

**Conclusions:**

Thus, our study demonstrates that CCRS expands the design ideas and RNA knockdown capabilities of Cas13 technology and has the potential to be used in disease treatment.

**Supplementary Information:**

The online version contains supplementary material available at 10.1186/s13059-023-02852-w.

## Introduction

Gene silencing technology is critical for the functional analysis of genes [1, 2]. With the rise of non-coding RNA research [3], RNA knockdown technology has become increasingly important. Antisense RNAs, ribozymes, and short hairpin RNAs (shRNAs) can be designed to bind specifically to any chosen target RNA, and this binding can block expression of the gene of interest [4, 5]. Although well-established, these traditional technologies still have many deficiencies and bottlenecks that prevent further development [6]. Antisense RNA refers to an RNA molecule that has a complementary sequence to the target RNA and participates in the regulation of gene expression by base pairing and binding with the target RNA. However, in most cases, the gene suppression effect of antisense RNA is very weak [7]. Natural or in vitro-evolved ribozymes are RNA molecules that possess the ability to catalyze specific biochemical reactions. Their most common activities include the cleavage or ligation of RNA and DNA and the formation of peptide bonds [8]. In order to further enhance the inhibitory effects of antisense RNAs, self-cleaving hammerhead ribozymes with fast cleavage kinetics have been designed to be fused with antisense RNA [9], leading to the specific and targeted degradation of the target RNA molecule. However, RNA molecules often form very complex secondary structures and may be coated with proteins or ribosomes, making it difficult for antisense RNA-mediated ribozymes to bind to the target sequence [10]. To overcome this, the construction of a library to identify optimal target sites for antisense-mediated gene inhibition is required. RNA interference (RNAi) is the highly efficient degradation of homologous mRNA induced by double-stranded small interfering RNA (siRNA) [11]. siRNAs processed in the cell combine with Ago2 and other proteins to form an RNA-induced silencing complex (RISC), resulting in the efficient degradation of target RNAs. Since the use of RNAi technology can often achieve excellent gene knockdown effects, this technology has been widely used in the field of gene therapy for exploring gene function in infectious diseases and malignant tumors [12]. However, this technology may have large off-target effects and certain cytotoxicity, which limits its wide application [13]. The effect of knocking down non-coding RNAs located in the nucleus is also not ideal, because RNAi happens in the cytoplasm.

In recent years, CRISPR/Cas9 has emerged as a powerful and convenient DNA editing technology [14]. In 2017, Abudayyeh et al. [15] discovered a new Cas protein Cas13a, which can target RNA for cleavage. Since then, the RNA-targeting molecules Cas13b (Cas13b1, Cas13b2 and Cas13bt), Cas13c, Cas13d, Cas13x, and Cas13y have also been discovered [16, 17]. Cas13 enzymes have an RNAse activity that is mediated by two HEPN domains and a CRISPR RNA (crRNA) maturation activity that maybe mediated by the HEPN2 and Helical-1 domains [18]. Because of their unique RNA-targeting capabilities, Cas13 family proteins have advantages in the detection and treatment of specific diseases and thus have become a major research focus in the field [19]. Before the development of CRISPR technology, RNAi was the most simple and effective way to regulate gene expression in cells, even though off-target effects were often reported. One of the major benefits of Cas13 is that it is potentially more target-specific. Furthermore, the CRISPR/Cas13 system is not endogenous to mammalian cells and thus is unlikely to disrupt the natural post-transcriptional network in the cell [20]. In contrast, RNAi uses an endogenous mechanism to carry out gene knockdown. The most direct application of Cas13 protein is to induce RNA silencing. At present, Cas13a, Cas13b, Cas13d, and Cas13x have been shown to interfere with RNA in mammalian cell lines to achieve gene silencing [16, 21]. Among them, the Cas13d family protein CasRx discovered in 2018 is considered to be an effective Cas13 protein to use in future applications due to its small size, high efficiency, and minimal protospacer flanking site (PFS) preference [22]. Some studies have suggested that compared with RNAi technology, CasRx-mediated gene silencing has a higher specificity and knockdown efficiency [22, 23]. Other studies have demonstrated that the RNA silencing effects of CasRx and shRNA are only comparable [24–26], suggesting that the system has room for further optimization.

Since CRISPR has the ability to specifically target DNA or RNA sequences, it can be used as a molecular scaffold to bind different functional molecules [27]. Modifying the CRISPR system through rational design can enable it to have biological functions that it did not originally possess. One conceivable method is to fuse the functional domains of various proteins with the Cas protein [28], so that DNA can be transcriptionally regulated and RNA can be base-edited or epigenetically modified [20, 21]. An alternative method that is used more frequently in catalytically inactive dead Cas9 (dCas9) and dCas12 systems involves integrating some non-coding RNA functional elements into the guide RNA [29]. This not only retains the ability of the guide RNA to bind the target sequence and the Cas protein but also introduces new functions such as binding to intracellular proteins through the newly fused non-coding RNA elements.

Fusion of the Cas protein with a functional domain will increase the size of the transgene required for expression [30]. Since many gene therapy vectors, such as adeno-associated virus (AAV), have certain capacity limitations, the transgene size may not be conducive to the development for gene therapy [31]. Therefore, an alternative strategy is to use non-coding RNA elements, which have shorter sequences but significant functions. For example, ribozymes have a very high self-cleavage activity in mammalian cells. In antisense RNA-mediated ribozyme technology, the 5′-end of the RNA is the antisense sequence that binds to the target, and the 3′-end is the ribozyme. In contrast, in the guide RNA of the Cas13 system, the 5′-end is a hairpin structure that binds to the Cas13 protein, and the 3′-end is an antisense RNA [32]. Thus, we hypothesized that fusion of the crRNA of CasRx and the antisense ribozyme may not only retain the ability of crRNA to bind CasRx protein and target RNA, but also enhance the RNA knockdown ability of CasRx through the RNA cleavage ability of ribozymes.

In this study, we describe a new guide RNA engineering strategy for CasRx to enable additional cutting of target RNAs by fusing the guide RNA of CasRx to an antisense ribozyme and test the ability of this approach in RNA knockdown and cancer gene therapy. We show that the CasRx-crRNA-Ribozyme system (CCRS) has much better RNA knockdown efficiency on both mRNAs and non-coding RNAs than those of the traditional methods, including CasRx, shRNA, and ribozyme. We then applied this strategy to the targeted inhibition of tumor cells both in vitro and in vivo, which also showed a better therapeutic potential than existing strategies.

## Results

### Design and construction of CCRS

**Fig. 1:**
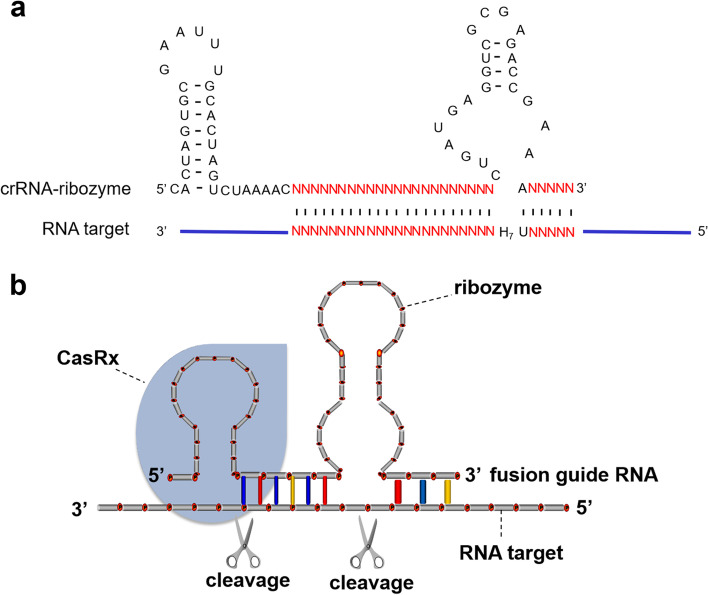
Design and construction of the CasRx-crRNA-Ribozyme system (CCRS). **a** Design of the fusion guide RNA construct of CCRS. The CasRx guide RNA has a 5′-scaffold sequence in front of the antisense RNA sequence, and the ribozyme shares the same 5′-antisense RNA with crRNA. The fusion guide RNA has scaffolds for CasRx and ribozyme, enabling RNA cleavage with both CasRx and ribozyme. H at position 7 designates A, C, or U. N indicates any nucleotide. **b** Schematic overview of the design CCRS strategy. CCRS would recruit both CasRx and ribozyme to its target transcript and induce double cleavages, thus enhancing the RNA knockdown efficiency of CasRx

First, we compared the guide RNA composition of the CRISPR/CasRx and the antisense ribozyme systems. In the CasRx system, the crRNA is composed of a short hairpin structure for binding CasRx protein, and a target-specific antisense RNA fused at the 3*′*-end of the crRNA. In the antisense RNA-mediated ribozyme system, the guide RNA includes a hammerhead ribozyme with a 5*′*-antisense RNA and a 3*′*-antisense RNA. In other words, the arrangement of CasRx crRNA and ribozyme guide RNA in the core functional domain and RNA recognition domain is reversed. From these features, we constructed a fusion guide RNA by connecting the 3*′*-end of the crRNA of CasRx to the 5*′*-end of the ribozyme (Fig. 1a), with the expectation that the resultant fusion guide RNA would recruit both CasRx and ribozyme to its RNA target and induce double cleavages (Fig. 1b). With the help of the catalytic core cassette of ribozyme, this was expected to further enhance the RNA degradation ability of CasRx.

### Targeting luciferase reporter gene with CCRS

**Fig. 2:**
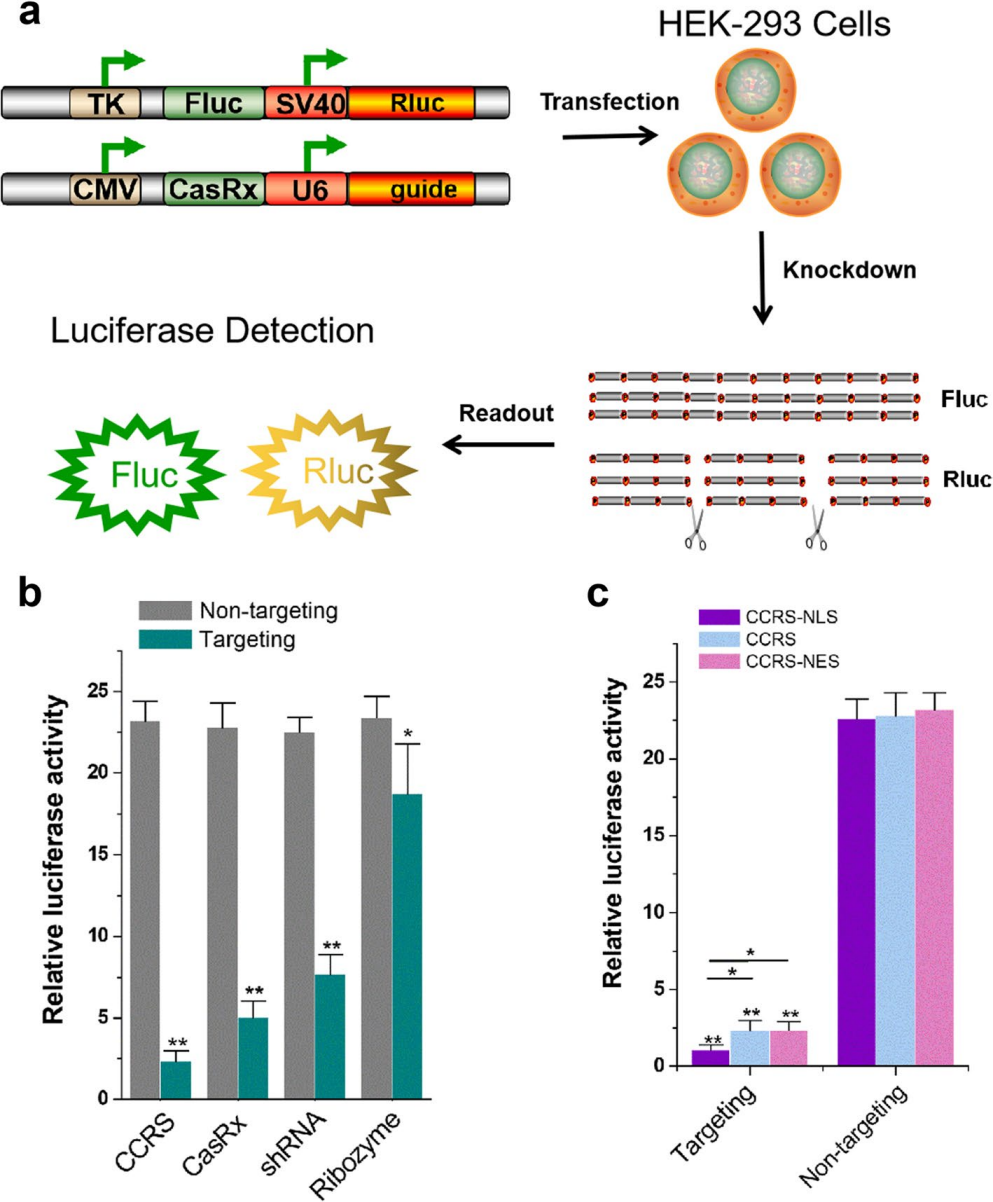
CCRS is capable of luciferase reporter transcript knockdown. **a** Schematic of the dual-luciferase reporter system used to evaluate RNA knockdown efficiency of CCRS. **b** Knockdown of Renilla luciferase (Rluc) using CCRS and other existing technologies. **c** Knockdown of Renilla luciferase (Rluc) using CCRS, CCRS-NLS, and CCRS-NES. NLS, nuclear localization signal. NES, nuclear export signal. Results are shown as the mean ± SD. Each experiment was performed in triplicate five independent times. *p value < 0.05, and **p value < 0.01, relative to the control using a two-tailed *t*-test

We next evaluated the in vivo cleavage ability of CCRS in mammalian cells, by using a dual luciferase reporter system, which expresses both Renilla luciferase (Rluc) and firefly luciferase (Fluc) driven by different promoters on a single vector. Rluc was chosen as the CCRS target by designing the fusion guide RNA and Fluc was treated as the internal control (Fig. 2a). The CCRS expression vector and dual-luciferase construct were co-transfected into HEK293 cells, and the luciferase activity was measured at 48 h post-transfection. Position-matched CasRx-crRNA, antisense ribozyme, and shRNA driven by the same U6 promoter were selected as positive controls and were also transfected into HEK293 cells and tested under the same conditions. The results indicated that CCRS efficiently knocked down Rluc activity by up to 90% relative to a non-targeting control guide (Fig. 2b). In contrast, CasRx and shRNA produced moderate inhibition of Rluc levels of 78% and 66%, respectively. The effect of antisense ribozyme is the weakest, with only 20% inhibition, indicating that antisense RNA has a poor binding ability to Rluc mRNA. In order to further confirm that the inhibition of luciferase activity is caused by mRNA knockdown, we also used qRT-PCR to detect the expression level of Rluc mRNA at 48 h after transfection, and the result was consistent with that of the dual luciferase assay (Additional file 1: Fig. S1a). We next fused the nuclear localization signal (NLS) with CasRx and found that NLS can further enhance the inhibitory effect of CCRS on Rluc (Fig. 2c) compared to CCRS without NLS and CCRS with the nuclear export signal (NES). mRNA levels detected by qRT-PCR also confirmed this observation (Additional file 1: Fig. S1b).

Since CasRx has the ability to process poly crRNAs [22], we wanted to rule out the possibility that CasRx cleaves crRNA-ribozymes in the CCRS system. To do this, we designed probes and amplification primers based on the sequences of wild-type CasRx crRNA and crRNA-ribozyme in CCRS and detected their relative expression levels by qRT-PCR. We found that CasRx had no effect on crRNA-ribozyme and wild-type crRNA expression levels (Additional file 1: Fig. S2a). Furthermore, the biochemical cleavage reaction revealed that the crRNA-ribozyme remained intact in the presence of CasRx (Additional file 1: Fig. S2b). To further validate the cleavage mechanism of CCRS, we introduced point mutations into the hammerhead ribozyme to inactivate ribozyme activity [33], while a dead version of CasRx [22] that was unable to cleave RNA was used as another control. The knockdown efficiency of CCRS was significantly reduced by using either the dead version of the ribozyme or dCasRx (Additional file 1: Fig. S3), suggesting that CCRS utilizes CasRx and the ribozyme simultaneously to cleave the targets. We also tried to fuse three different versions of hammerhead ribozymes [9, 33, 34] into the crRNA of the CasRx system and compared the knockdown efficiency of the various versions of the CCRS system. Although they were all able to reduce the expression of the target gene to varying degrees, the version we originally used had the highest knockdown efficiency (Additional file 1: Fig. S4). Thus, we used the original ribozyme CCRS system for subsequent experiments.

These results suggest that CCRS can knock down foreign reporter genes more effectively than existing strategies.

### Targeting endogenous mRNAs and non-coding RNAs with CCRS

**Fig. 3:**
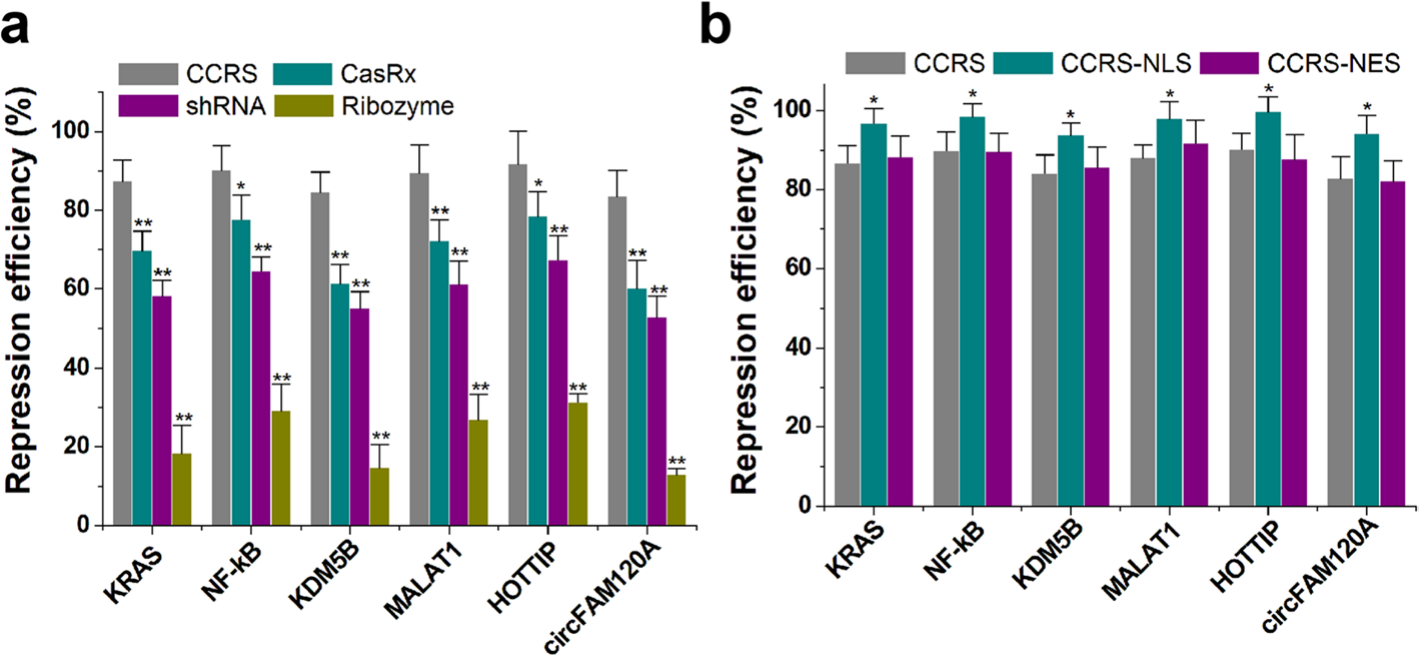
Targeting endogenous transcripts with CCRS. **a** Knockdown of six endogenous transcripts following transfection of cells with CCRS and other existing technologies was assayed using qRT-PCR. **b** Knockdown of six endogenous transcripts using CCRS, CCRS-NLS, and CCRS-NES. NLS, nuclear localization signal. NES, nuclear export signal. Repression efficiency values are the decline percentages (%) of the transcript expression levels relative to the corresponding negative control. The results are shown as the mean ± SD. Each experiment was performed in triplicate five independent times. Each error bar indicates the variation between the means of five independent experiments. *p value < 0.05, and **p value < 0.01, relative to the control using a two-tailed *t*-test

We next compared the ability of CCRS constructs to knockdown endogenous transcripts in HEK293 cells. We selected three coding RNAs (KRAS, NF-κB, KDM5B) and three non-coding RNAs (MALAT1, HOTTIP, circFAM120A). The relative transcript level was measured at 48 hours post-transfection by qRT-PCR. We observed varying levels of knockdown, and found that CCRS targeted all mRNAs (KRAS, NF-κB, KDM5B), linear lncRNAs (MALAT1, HOTTIP), and circular RNA (circFAM120A) more efficiently than wild-type CasRx, shRNA, and antisense ribozyme (Fig. 3a). We also conducted the same experiment in primary cultured fibroblasts and further confirmed the above results (Additional file 1: Fig. S5). We then found that addition of an NLS further enhanced the inhibitory effect of CCRS on endogenous transcripts (Fig. 3b). Thus, CCRS-NLS was used for subsequent experiments. These results suggest that CCRS can be used to more effectively inhibit the expression of both coding and non-coding transcripts than existing approaches.

### Targeting specificity of CCRS

**Fig. 4:**
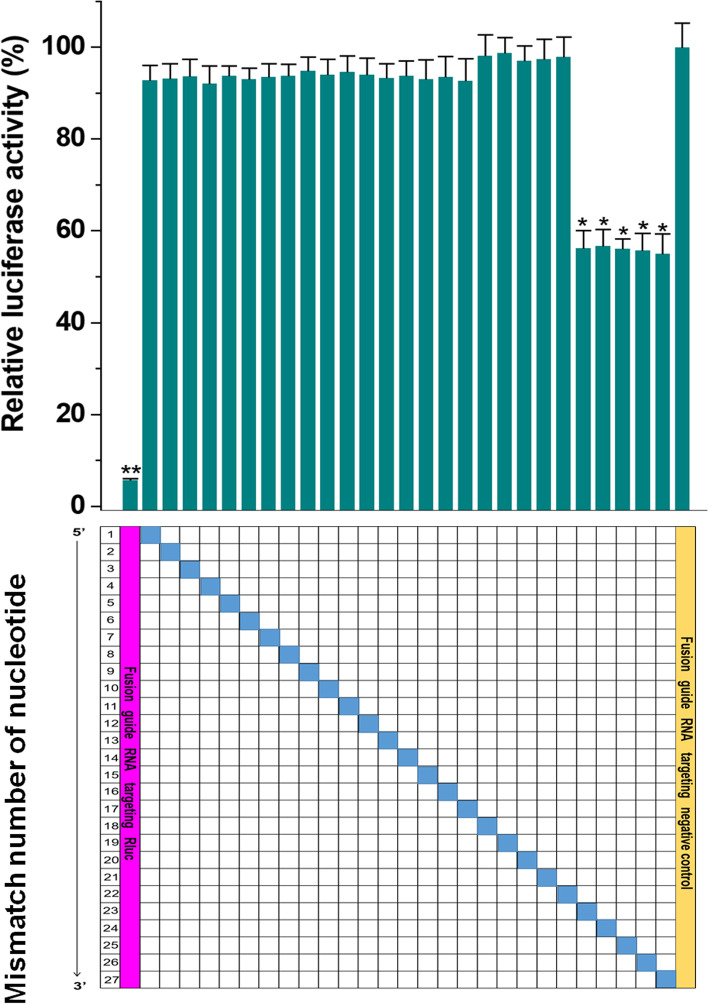
Evaluation of CCRS knockdown specificity. Knockdown of Rluc was evaluated with the fusion guides of CCRS containing single mismatches at varying positions across the antisense sequence. Data are expressed as the mean ± SD. Each experiment was performed in triplicate five independent times. Each error bar indicates the variation between the means of five independent experiments. *p value < 0.05, and **p value < 0.01, relative to the control using a two-tailed *t*-test

To determine the specificity of CCRS at targeting RNA transcripts, we addressed the sensitivity of the fusion guide RNA to mismatches. To assess mismatch tolerance, we investigated targeting effects when introducing single mismatches into the duplex formed between the fusion guide RNA and the RNA target. The relative luciferase activity assay indicated that even one mismatch in the guide region (nucleotides 1–22) dramatically decreased CCRS knockdown efficiency (Fig. 4). Any single mismatch of nucleotides 23–27 seemed to eliminate the knockdown effect of the antisense ribozyme, while the most effect of CasRx might be retained. To test whether CCRS can induce the off-target collateral cleavage, RNA-denaturing gel electrophoresis of total RNA was performed and the results showed that both targeting crRNAs and nontargeting crRNAs did not induce ribosomal RNA degradation 48h after transfection (Additional file 1: Fig. S6). According to the results of RNA-seq, Rluc mRNA was the only transcript that was significantly inhibited by the CCRS system (Additional file 1: Fig. S7). Similar results were obtained for the same comparison on the endogenous gene KRAS (Additional file 1: Fig. S8). In addition, the mismatch sensitivity of crRNA was also tested using the wild-type CasRx system. Interestingly, while single mismatches could reduce the activity of guide RNAs of CasRx, they rarely eliminate it completely (Additional file 1: Fig. S9). The crRNAs were particularly sensitive to mismatches in nucleotides 15 to 21, which was consistent with the result of one previous study [32]. Taken together, these data show that CCRS is very sensitive to mismatches in general and may have a low off-target potential, consistent with previous studies of CasRx [22, 23].

### Simultaneous targeting of multiple endogenous RNAs with CCRS

**Fig. 5:**
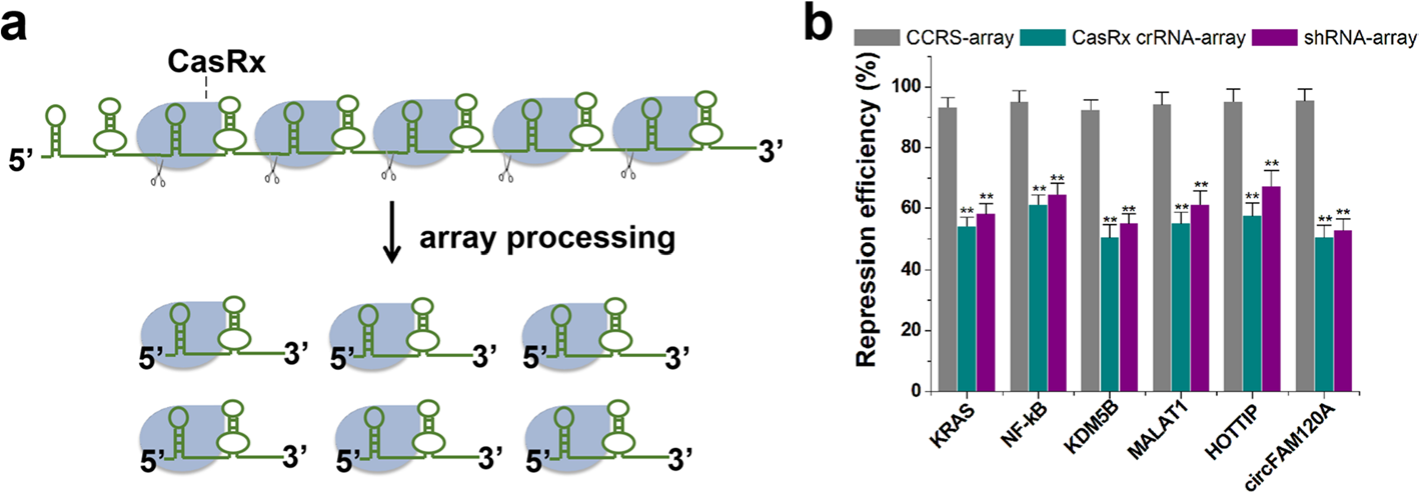
Multiplexed targeting of diverse human coding and non-coding transcripts with CCRS. **a** Multiple fusion guide RNAs targeting diverse target transcripts can be expressed as a single array and processed by CasRx protein into individual guide RNAs within the same cell. **b** qRT-PCR analysis of relative target RNA knockdown by CCRS-array, CasRx crRNA-array, and shRNA-array. Repression efficiency values were the decline percentages (%) of transcript expression levels relative to the corresponding negative control. Data are expressed as the mean ± SD. Each experiment was performed in triplicate five independent times. Each error bar indicates the variation between the means of five independent experiments. *p value < 0.05, and **p value < 0.01, relative to the control using a two-tailed *t*-test

Because CasRx is capable of processing its own CRISPR array, we next sought to extend the application of CCRS in a multiplexed targeting manner. We tested whether CCRS can be used to simultaneously target multiple transcripts (KRAS, NF-κB, KDM5B, MALAT1, HOTTIP, circFAM120A) in the same cell for the delivery of multiple targeting fusion guide RNAs in a simple single-vector system (Fig. 5a). We also sought to benchmark CCRS against established technologies, including CasRx and shRNA. Relative RNA levels were detected at 48 h post-transfection by qRT-PCR. As expected, we observed a decrease in RNA levels across all six endogenous transcripts (Fig. 5b). Unexpectedly, we observed a moderate decrease in wild-type CasRx efficiency when we deployed several guide RNAs in the same cells, which we attribute to a possible dilution effect. This phenomenon does not seem to exist in shRNA, as the RNA inhibition efficiency of multiple shRNAs in tandem driven by a single U6 promoter was not different from that of a single shRNA (Fig. 3b). Interestingly, CCRS outperformed shRNAs and CasRx in each case (Fig. 5b), exhibiting a median knockdown efficiency (for different target genes) of 94% compared to 60% for shRNA and 55% for CasRx after 48 h. We further extended the length (22 nt) of the crRNA of CasRx to make it consistent with the length (28 nt) of the CCRS antisense region, but the knockdown effect on these six transcripts was only slightly improved (Additional file 1: Fig. S10). Several reported target sites [22] (EGFR, EZH2, HRAS, NRAS, RAF1, and STAT3) were also employed to compare with CCRS and similar results were obtained (Additional file 1: Fig. S11a). We then constructed two crRNA arrays independently driven by the U6 promoter in the CCRS system, each targeting one of the 6-gene combinations described above. It was found that the knockdown effect of CCRS was reduced when the number of targets reached 12, but its effect was still higher than that of wild-type CasRx (Additional file 1: Fig. S11b). These data suggest that CCRS allows for targeting multiple transcripts at once and achieves a higher efficiency than traditional technologies.

### AAV-mediated delivery of CCRS into cancer cells

**Fig. 6:**
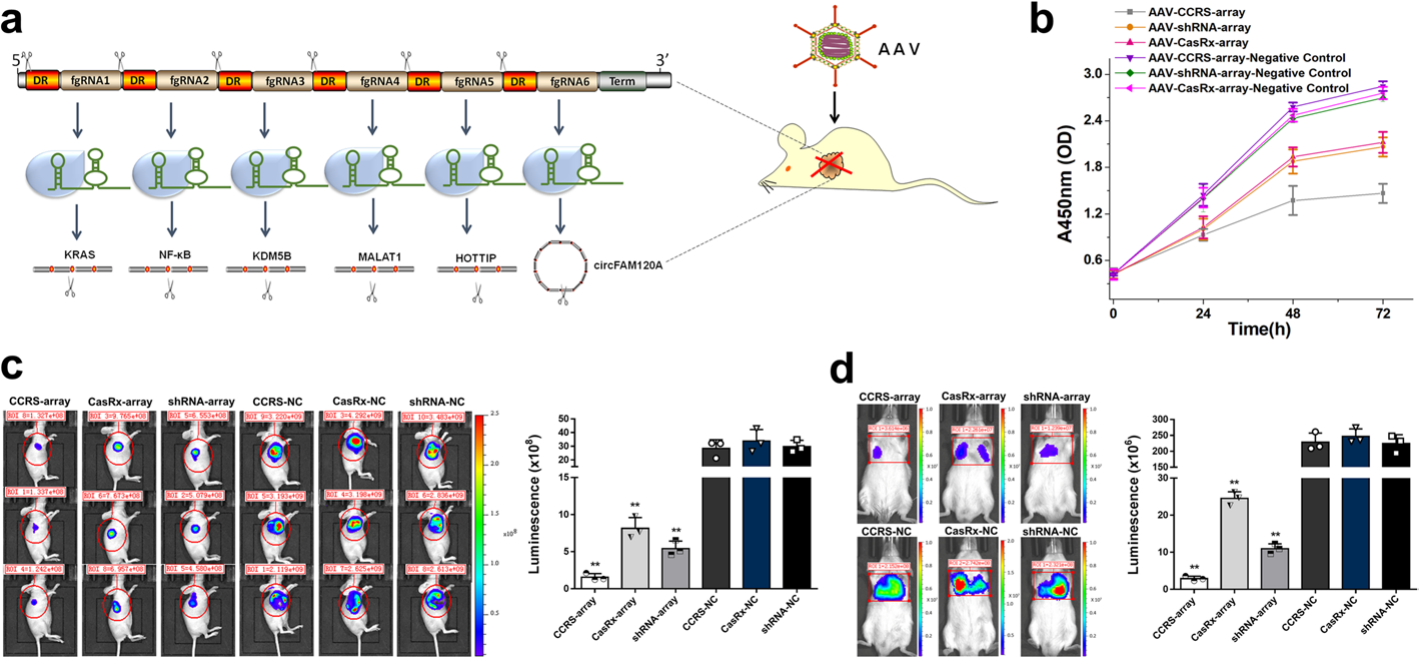
Multiple gene targeting and adeno-associated virus (AAV) delivery of CCRS in cancer cells. **a** A schematic diagram of CCRS delivered by AAV, which effectively inhibits tumors by targeting multiple oncogenes. DR, direct repeat. fgRNA, fusion guide RNA. **b** Growth curves of bladder cancer T24 cells treated with CCRS and other existing technologies. The curves of cell proliferation were compared using analysis of variance (ANOVA). The results at each time point are shown as the mean ± SD. Each experiment was independently performed in triplicate five independent times. Each error bar indicates the variation between the means of five independent experiments. **c** AAV-CCRS efficiently inhibited in vivo tumor growth. The luminescent signal intensities for AAV-CCRS, AAV-CasRx and AAV-shRNA and their corresponding negative controls are shown. Data are shown as the mean ± SD. **p < 0.01, between the groups, using a two-tailed t-test. **d** Quantification for bioluminescence imaging of a metastatic model. The luminescent signal intensities for AAV-CCRS, AAV-CasRx and AAV-shRNA are shown. Data are shown as the mean ± SD. **p < 0.01, between the groups, using a two-tailed t-test

Since CCRS allows for multiple endogenous transcripts to be simultaneously regulated, this opens up new possibilities for cancer gene therapy by targeting multiple oncogenic genes at once (Fig. 6a). Another core advantage of CCRS is the small size, which should permit efficient AAV packaging and delivery. To showcase the possibility of AAV-mediated CCRS delivery, we packaged the CCRS expression plasmid into the AAV delivery vehicle. We chose bladder cancer as a model and found that transduction of bladder cancer T24 cells with the generated AAV recapitulates the knockdown efficiency of CCRS on the six endogenous targets (Additional file 1: Fig. S12), confirming AAV-delivered CCRS is still functional and provides an approach towards clinical usage.

Next, we tested the anticancer effects of AAV-mediated CCRS on bladder cancer and compared the efficiency of CCRS with the existing approaches including CasRx and shRNA. Cell proliferation was determined at various time points using the CCK-8 assay. We found that CCRS resulted in approximately 70% inhibition of T24 cell proliferation, while CasRx and shRNA had a moderate affect (Fig. 6b). In the CCRS system, the 6-mer array also showed a stronger and more significant effect than individual guides and smaller arrays in inhibiting cell proliferation (Additional file 1: Fig. S13a). To rule out the possibility that the ability of CCRS to inhibit cell viability is caused by cytotoxicity of ribozyme, we treated T24 cells with AAVs expressing crRNA-ribozyme array without CasRx and observed no significant inhibitory effect on cell viability (Additional file 1: Fig. S13b). Consistent with these findings, CCRS also induced T24 cell apoptosis and mediated a significantly greater effect than CasRx and shRNA, as determined by caspase-3/ELISA (Additional file 1: Fig. S14). In addition, the wound healing assay indicated that CCRS attenuated migration of bladder cancer cells (Additional file 1: Fig. S15) and that CCRS outperformed CasRx and shRNA in suppressing T24 cell migration.

To further explore the potential applications of AAV-mediated CCRS, we conducted in vivo experiments with subcutaneous tumor models, where AAVs were injected directly into tumors ten days after inoculation of T24 cells expressing luciferase. We found that the volume, weight and fluorescence intensity of xenografted tumors injected with AAVs were decreased compared with that of the control group (Fig. 6c and Additional file 1: Fig. S16). In addition, CCRS outperformed CasRx and shRNA in suppressing tumor growth in vivo. The T24 cell line stably expressing luciferase was then used to develop an in vivo bladder cancer lung metastasis model. The telomerase reverse transcriptase (TERT) promoter, a tumor-specific promoter, was used to drive CCRS expression. After injecting AAVs through the tail vein, CCRS significantly suppressed the growth of lung metastases and was found to be more effective than CasRx and shRNA (Fig. 6d). Hematoxylin-eosin staining was then performed on the lung tissue to observe the metastases in the groups. CCRS indeed significantly reduced the number and size of pulmonary metastases, while CasRx and shRNA only partially exerted such effects (Additional file 1: Fig. S17).

Together, these results suggest that CCRS can be used to strongly inhibit the growth and metastasis of bladder cancer cells in vivo and achieves a higher efficiency than traditional technologies.

## Discussion

Although RNAs, including mRNA, lncRNA, and circular RNAs are abundant and play important and diverse roles in mammalian cells, the molecular tools available to regulate RNA expression are limited [35]. Several approaches, such as antisense RNAs, ribozymes, and RNAi, have been developed to study the biological roles of RNAs and target RNAs for therapeutic purposes. Each of these approaches has its own challenges in terms of efficiency, specificity, immunogenicity, toxicity, and delivery [36, 37]. Therefore, an efficient RNA knockdown strategy is still required. Recently, CRISPR/Cas13, a novel RNA-guided RNA-targeting CRISPR-Cas effector system was reported to target and cleave mRNAs, lncRNAs, and circular RNAs in cells [38]. All available Cas13-related proteins (LwaCas13a, PguCas13b, PspCas13b, RanCas13b, AdmCas13d, EsCas13d, and RfxCas13d) provided comparable levels of knockdown as RNAi. CasRx (also named RfxCas13d) exhibited excellent knockdown efficiency with less off-target effects on RNAs [39]. Apart from the complementarity between crRNA and target RNA, CasRx does not show a PFS preference and no obvious off-target effects have been detected in previous studies [40]. The efficiency of CasRx is dramatically decreased or even eliminated if 1–2 mismatches occur in the seed region of crRNA. A question worthy of in-depth study is whether the RNA knockdown function of CasRx can be optimized further and applied to gene therapy of diseases.

Here, we connected the 3′-end of the crRNA of CasRx to the 5′-end of the ribozyme, thus developing CCRS technology. Our results show that the RNA knockdown effect of CCRS is more effective than wild-type CasRx, ribozyme, and shRNA when targeting luciferase reporter genes and endogenous RNAs. Examining single base substitutions revealed that CCRS not only improves the RNA cleavage efficiency of CasRx but also maintains the targeting specificity, thereby indicating that CCRS is a reliable RNA knockdown strategy with more application potential than previous strategies.

It is important to note that although CasRx can be used to simultaneously target multiple transcripts, its knockdown efficiency decreases with an increasing number of guides targeting different transcripts. This phenomenon may be related to the dilution effect, because the CasRx protein not only processes the precursor crRNA but also binds and cleaves a variety of different transcripts. Indeed, increasing the number of knockdown targets to 12 slightly reduced the effectiveness of CCRS.

Our results show that the performance of CCRS in this case is more effective than that of wild-type CasRx, which may be attributed to the ribozyme activity and the longer complementary antisense sequence of CCRS that enhances the binding of CasRx to target RNA. However, the performance of CasRx was only slightly improved when we used crRNA with the same length as the antisense sequence of CCRS, suggesting that the ribozyme and antisense sequences may synergistically promote the binding of RNA targets. The knockdown efficiency of CCRS was significantly affected by using either the dead version of the ribozyme or dCasRx, which further suggest that CCRS should utilize both CasRx and ribozyme activities to cleave the targets.

Next, we used bladder cancer as a model to evaluate the anticancer effects of CCRS targeting multiple genes both in vitro and in vivo. We selected several endogenous targets that are commonly used as cancer treatment targets. CCRS showed a higher anticancer effect than CasRx, ribozyme, and shRNA, consistent with our findings from the in vitro gene knockdown studies. Compared with the CasRx system, CCRS contains only one extra ribozyme and, therefore, can be easily loaded into AAV for gene therapy. This demonstrates the feasibility of CCRS for the treatment of cancers or other diseases.

The antisense ribozyme can also be applied to guide RNAs of other Cas13 enzymes besides CasRx. Many Cas13 variants, such as Cas13bt, Cas13x, and Cas13y, have been shown to be very efficient. As Cas13bt, Cas13x, and Cas13y are compact Cas13 members, they may have advantages compared to CasRx [16, 17, 41, 42]. Indeed, if new nucleases of the Cas13 family with higher RNA knockdown efficiency are found, the CCRS strategy will have better application prospects.

CCRS may also be used to regulate gene expression in bacteria, due to the high biological activity of CasRx nuclease and ribozyme in prokaryotes [43, 44]. As RNAi is non-functional in prokaryotic systems [45], CCRS may be a promising tool in the regulation of prokaryotic gene expression in the future.

Although our results suggested that CCRS is conceptually simpler than the independent CasRx/ribozyme system to design, the target sequences of CasRx and ribozyme in the CCRS are stringently associated and may be selected carefully. In order to optimize the effectiveness of the CCRS system, further research is needed to determine how to select the best targets.

## Conclusions

In conclusion, this study describes an alternative RNA targeting tool that can knock down gene expression with higher efficiency than existing approaches. CCRS can be used not only for gene function research but can also be potentially used in gene therapy of diseases.

## Methods

### Cell lines and cell culture

The HEK-293, HEK-293T, and T24 cell lines used in this study were purchased from American Type Culture Collection (ATCC). Normal human primary fibroblasts derived from the epidermis were kindly provided by Dr. T. Chen (Shantou University, Shantou, China). All cell lines were grown in DMEM medium supplemented with 10% fetal bovine serum (Invitrogen, Carlsbad, CA, USA) in the presence of 5% CO_2_. Cell cultures were also confirmed to be free of Mycoplasma contamination by using 16S rRNA-based Mycoplasma group-specific polymerase chain reaction (PCR).

### Construction of plasmids

The backbone plasmid expressing human codon-optimized CasRx protein was purchased from Addgene (# 109049). The shRNA-expressing plasmids were purchased from Syngentech Co., Ltd. (Beijing, China). crRNA sequences and crRNA-ribozymes that exactly match the corresponding shRNA sequences were designed. The cDNA sequences for crRNA, ribozyme, crRNA-ribozyme, and shRNA were then synthesized and inserted into corresponding backbone plasmids digested with restriction endonucleases. All vectors were transformed into One Shot TOP10 Chemically Competent *E. coli* cells. The desired expression clones were identified using PCR amplification and electrophoresis and then confirmed with Sanger sequencing. The sequence information of the related elements is shown in Additional file 2: Table S1.

### DNA transfection into cells

HEK-293, HEK-293T, and T24 cells were cultured in plates until they reached 70% confluence. Cells were transiently transfected with the constructed plasmids using Lipofectamine 3000 (Invitrogen) according to the manufacturer’s protocol.

### Dual-luciferase reporter assay

Both the Renilla luciferase and firefly luciferase activities were measured in a 1.5-ml Eppendorf tube using the Promega Dual-Luciferase Reporter Assay kit (Promega E1980) according to the manufacturer’s protocol 48 h after DNA transfection. The relative luciferase activity was calculated as the Renilla luciferase value normalized to that of the firefly luciferase value. The assays were performed in triplicate and experiments were repeated five times.

### RNA extraction and real-time quantitative PCR

Total RNAs were isolated from cells using TRIzol reagent (Invitrogen) according to the manufacturer’s protocol. The concentration and purity of total RNA were measured using UV spectrophotometric analysis at 260 nm. cDNAs were synthesized using the Superscript III First Strand Synthesis System (Invitrogen). Real-time quantitative reverse transcription PCR (qRT-PCR) was performed using a SYBR Green PCR Master Mix (Invitrogen). GAPDH was chosen as the endogenous control. PCR mixtures were prepared according to the manufacturer’s protocol and amplification was performed under the following PCR parameters on an ABI PRISM 7300 Fluorescent Quantitative PCR System (Applied Biosystems, Foster City, CA, USA): 40 cycles of 15 s at 95 °C, 20 s at 55 °C, and 30 s at 70 °C. All primer sequences are shown in Additional file 3: Table S2. Expression fold changes were calculated using the 2^−△△ct^ method.

### RNA-denaturing gel electrophoresis

Total RNAs were obtained using TRIzol reagent (Invitrogen) according to the manufacturer’s protocol. One gram of agarose was heated in 72 ml water until dissolved and then cooled to 60 °C. Ten milliliters of 10X MOPS running buffer and 18 ml 37% formaldehyde (12.3 M) were then added to prepare the gel. The gel was prerun for 10 min at 80 V, and 5 μg of total RNA was then loaded and separated until the bromophenol blue migrated 3 cm into the gel. The gel was visualized on a UV transilluminator.

### Analysis of the specificity of CCRS

Total RNA was isolated, mixed with oligo (dT)^25^ Dynabeads, and mRNA was purified according to the manufacturers’ instructions (Invitrogen). The purified mRNA was first fragmented by using divalent cations before being subjected to library preparation. Adapters were ligated to fragmented RNAs by using truncated T4 RNA ligase 2 (NEB) according to the manufacturer’s instructions, and RNA was reverse transcribed to DNA by using SuperScript® III (Thermo Scientific, Scotts Valley, CA, USA) and circularized by using Circligase™ (EpiBio/Illumina Madison, WI, USA). Barcodes were added by PCR using Phusion® polymerase (Thermo Scientific). DNA libraries were sequenced on an Illumina HiSeq 2500 (Illumina). Reads were processed by Cufflinks v2.1.1 and fold changes were then calculated according to fragments per kilobase of transcript per million mapped reads (FPKM) values.

### AAV packaging, purification and titer detection

The pAAV packaging plasmid, pHelper plasmid, and pAAV plasmid were co-transfected into HEK-293T cells using Lipofectamine 3000. The culture supernatants were collected at 48 hours after plasmid transfection, concentrated, and used as virus stocks for subsequent AAV infection experiments. The AAV titer was calculated by qPCR using 2× EvaGreen Master Mix (Syngentech).

### Cell proliferation assay

Cell proliferation was assayed using the Cell Counting Kit-8 (CCK-8) (Beyotime, Shanghai, China) according to the manufacturer’s instructions. At various time points (0, 24, 48, or 72 h) post-transduction, 10 μl of CCK-8 reagent was added to each well of the 96-well plates, and the cells were incubated for an hour. Absorbance was read at a wavelength of 450 nm using a microplate reader (Bio-Rad, Hercules, CA). The assays were performed in triplicate and experiments were repeated five times.

### Cell apoptosis assay

According to the manufacturer’s protocol, cell apoptosis was determined by the caspase-3/ELISA (enzyme-linked immunosorbent assay) assay (Cusabio, China). Caspase-3 is a marker for inflammation and apoptosis signaling. The experiment for each sample was repeated five times independently.

### Cell migration assay

Cell migration was determined by the wound healing assay. Briefly, cells were seeded into 12-well plates at equal density and cultured to 80% confluency. Artificial gaps were generated using a sterile pipette tip. Areas of wound were marked and photographed using a digital camera system. Cell migration distance (mm) was calculated using the software program HMIAS-2000. Each experiment was repeated five times.

### Tumor xenografts

The mice were housed under standard laboratory conditions. BALB/c-Nude mice were randomly assigned into either the experimental group or control groups (five mice for each group). Bladder cancer T24 cells (5 × 10^7^) were hypodermically injected on the backs of BALB/c-Nude mice. Next, intratumoral injection of AAV (100 μl, 2 × 10^11^ vp/ml) was performed. Tumor volumes were calculated using the formula: *V* = *L* × *W*^2^/2, where *L* is the length and *W* is the width of the tumor. The mice were sacrificed and tumors were removed at the end of the experiment.

For in vivo imaging experiments, BALB/c-Nude mice were randomly assigned into either the experimental group or control groups (three mice for each group). Bladder cancer T24 cells (5 × 10^7^) were hypodermically injected on the backs of BALB/c -Nude mice, and then a single injection of AAV (100 μL, 2 × 10^11^ vp/mL) via tail vein was conducted 10 days after inoculation. Four weeks later, mice were anaesthetized with isoflurane and injected with D-luciferin sodium salt (150 mg/kg) intraperitoneally. Subcutaneous tumor was then monitored by an in vivo imaging system, Xenogen IVIS (PerkinElmer, MA, USA).

### Experimental metastasis mouse model

T24 bladder cancer cells stably expressing luciferase (1 × 10^5^) were suspended in 200 μL PBS and injected into the lateral tail veins of 5-week-old male B-NDG mice (BIOCYTOGEN, Beijing, China). Four weeks later, mice were anaesthetized with isoflurane, and D-luciferin sodium salt (150 mg/kg) was injected intraperitoneally. Bladder cancer cells were detected with an in vivo imaging system, Xenogen IVIS (PerkinElmer, MA, USA). The total flux in photons per second was calculated for the lung region using Living Image 4.3.1 (PerkinElmer/Caliper).

### H&E staining of lung tissues

Mouse lung tissues were fixed in 10% formalin and dehydrated in ethanol. Paraffin embedding, sectioning, and hematoxylin and eosin staining were performed according to the manufacturer’s procedures, and then slides were imaged on a Nikon Ci-L bright field microscope.

### Statistical analyses

Data were expressed as the mean ± standard deviation (SD). Significance tests were performed using the SPSS version 20.0 software (SPSS, Chicago, IL, USA). Statistical significance was determined using Student’s *t*-test or ANOVA and assigned at *p* < 0.05.

## Supplementary Information

**Additional file 1: Figure S1.** The expression level of Renilla luciferase (Rluc) mRNA in HEK293 cells. **Figure S2.** Detection of the potential influence of CasRx on the engineered crRNAs. **Figure S3.** Knockdown of Renilla luciferase (Rluc) using CCRS, CCRS-mutants and other related technologies. **Figure S4.** Comparison of knockdown effects of different versions of hammerhead ribozymes in the CCRS system. **Figure S5.** Targeting endogenous transcripts with CCRS in primary cultured cells. **Figure S6.** RNA-denaturing gel electrophoresis examining RNA integrity. **Figure S7.** Analysis of the specificity of CCRS-mediated knockdown. **Figure S8.** Evaluation of CCRS knockdown specificity on endogenous gene. **Figure S9.** Evaluation of CasRx knockdown specificity on luciferase gene. **Figure S10.** qRT-PCR analysis of relative target RNA knockdown by CasRx and shRNA. **Figure S11.** Targeting other endogenous transcripts with CCRS. **Figure S12.** qRT-PCR analysis of relative target RNA knockdown by AAV-CCRS and other existing technologies. **Figure S13.** The inhibition rate (%) of T24 cell proliferation. **Figure S14.** ELISA assay on Caspase-3 activity in T24 cells treated by AAV-CCRS and other existing technologies. **Figure S15.** AAV-CCRS efficiently inhibited T24 cell migration. **Figure S16.** AAV-CCRS efficiently inhibited in vivo tumor growth. **Figure S17.** Histopathological inspection of the mouse lungs treated with AAVs.

**Additional file 2: Table S1.** The cDNA sequences of the engineered elements used in this study.

**Additional file 3: Table S2.** Primer sequences used in real-time quantitative PCR.

**Additional file 4.** Review history.

## Data Availability

The data generated or analyzed during this study are included in this article and its additional files. The data supporting the findings in the main text can be found in Figures S1, S2, S3, S4, S5, S6, S7, S8, S9, S10, S11, S12, S13, S14, S15, S16 and S17. The sequences of elements used in this study are listed in Tables S1 and S2. The RNA-seq data have been submitted to the GenBank Sequence Read Archive under the accession number PRJNA891500 [46].
