# Supplementary material for: Enhanced RNA knockdown efficiency with engineered fusion guide RNAs that function with both CRISPR-CasRx and hammerhead ribozyme
Source: Genome Biol. 2023 Jan 17;24:9. doi: 10.1186/s13059-023-02852-w (PMC9843992; doi:10.1186/s13059-023-02852-w)
Supplement: Supplementary file 1 — Additional file 1: Figure S1. The expression level of Renilla luciferase (Rluc) mRNA in HEK293 cells. Figure S2. Detection of the potential influence of CasRx on the engineered crRNAs. Figure S3. Knockdown of Renilla luciferase (Rluc) using CCRS, CCRS-mutants and other related technologies. Figure S4. Comparison of knockdown effects of different versions of hammerhead ribozymes in the CCRS system. Figure S5. Targeting endogenous transcripts with CCRS in primary cultured cells. Figure S6. RNA-denaturing gel electrophoresis examining RNA integrity. Figure S7. Analysis of the specificity of CCRS-mediated knockdown. Figure S8. Evaluation of CCRS knockdown specificity on endogenous gene. Figure S9. Evaluation of CasRx knockdown specificity on luciferase gene. Figure S10. qRT-PCR analysis of relative target RNA knockdown by CasRx and shRNA. Figure S11. Targeting other endogenous transcripts with CCRS. Figure S12. qRT-PCR analysis of relative target RNA knockdown by AAV-CCRS and other existing technologies. Figure S13. The inhibition rate (%) of T24 cell proliferation. Figure S14. ELISA assay on Caspase-3 activity in T24 cells treated by AAV-CCRS and other existing technologies. Figure S15. AAV-CCRS efficiently inhibited T24 cell migration. Figure S16. AAV-CCRS efficiently inhibited in vivo tumor growth. Figure S17. Histopathological inspection of the mouse lungs treated with AAVs. [file 13059_2023_2852_MOESM1_ESM.doc]

**
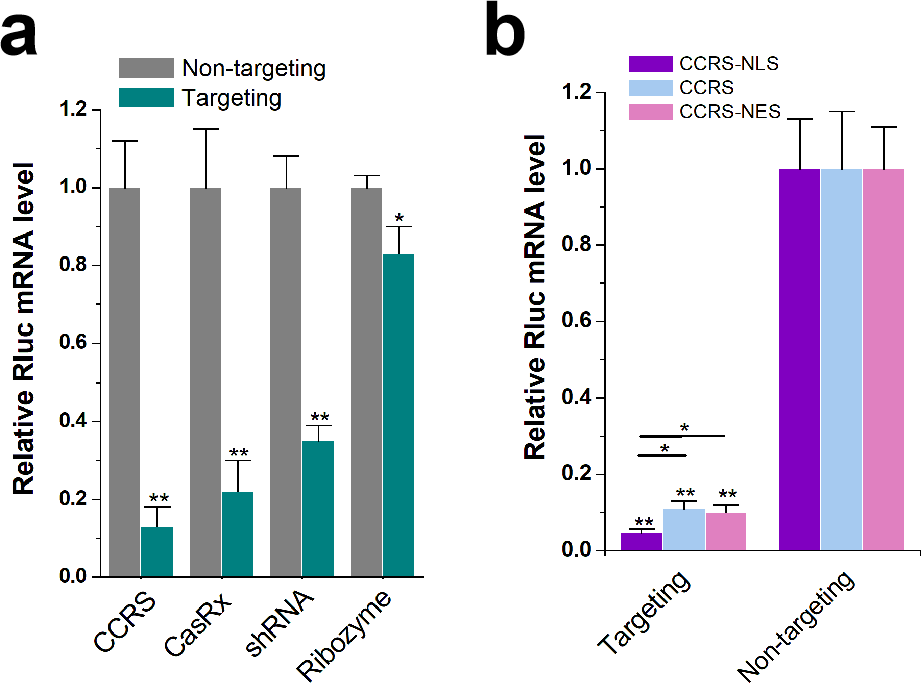
**

**Figure S1.** **The expression level of Renilla luciferase (Rluc) mRNA in HEK293 cells. (a)** Knockdown of Rluc mRNA using CCRS and other existing technologies. **(b)** Knockdown of Rluc mRNA using CCRS, CCRS-NLS and CCRS-NES. NLS, nuclear localization signal. NES, nuclear export signal. The relative expression level of Rluc was determined by qRT-PCR. Firefly luciferase (Fluc) mRNA was used as the internal control. Each experiment was performed in triplicate for five independent times. Each error bar indicates the variation between the means of five independent experiments. *, p value < 0.05, and **, p value < 0.01, relative to the control using a two-tailed t test.


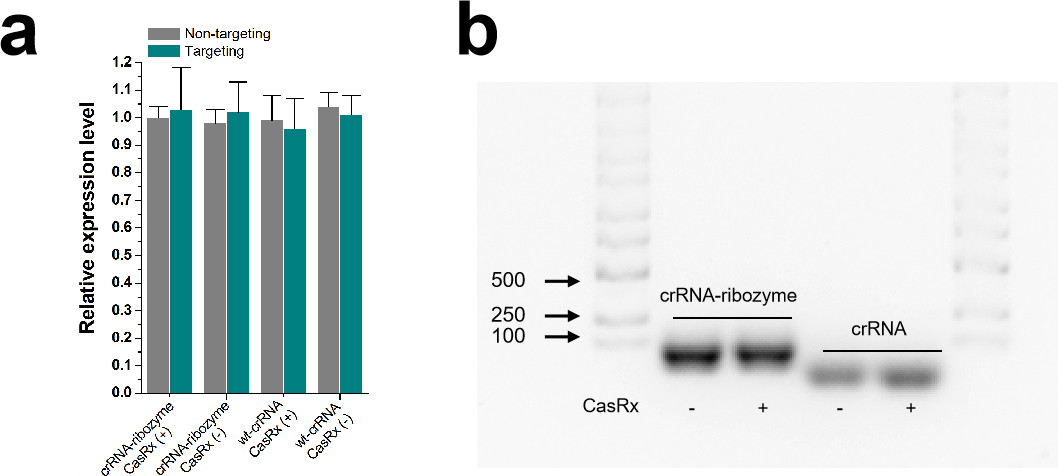


**Figure S2. Detection of the potential influence of CasRx on the engineered crRNAs. (a)** The expression level of crRNAs in HEK293 cells. The relative expression levels of crRNA-ribozyme and wild-type crRNAs were determined by qRT-PCR. GAPDH was used as the internal control. Each experiment was performed in triplicate for five independent times. Each error bar indicates the variation between the means of five independent experiments. **(b)** Addition of purified CasRx protein did not cleave the engineered crRNA-ribozyme.





**Figure S3. Knockdown of Renilla luciferase (Rluc) using CCRS, CCRS-mutants and other related technologies.** The knockdown effect of CCRS was higher than that of the CasRx crRNA+ribozyme group (which were driven independently by two U6 promoters). When the ribozyme was mutated, the knockdown efficiency of CCRS was comparable to that of wild-type CasRx. When CasRx was mutated, the knockdown efficiency of CCRS was higher than that of the wild-type ribozyme. In the absence of dCasRx transfection, the knockdown efficiency of the crRNA-ribozyme was comparable to that of the wild-type ribozyme. Results are shown as the mean ± SD. Each experiment was performed in triplicate five independent times. *, p value < 0.05, and **, p value < 0.01, relative to the control using a two-tailed t-test.


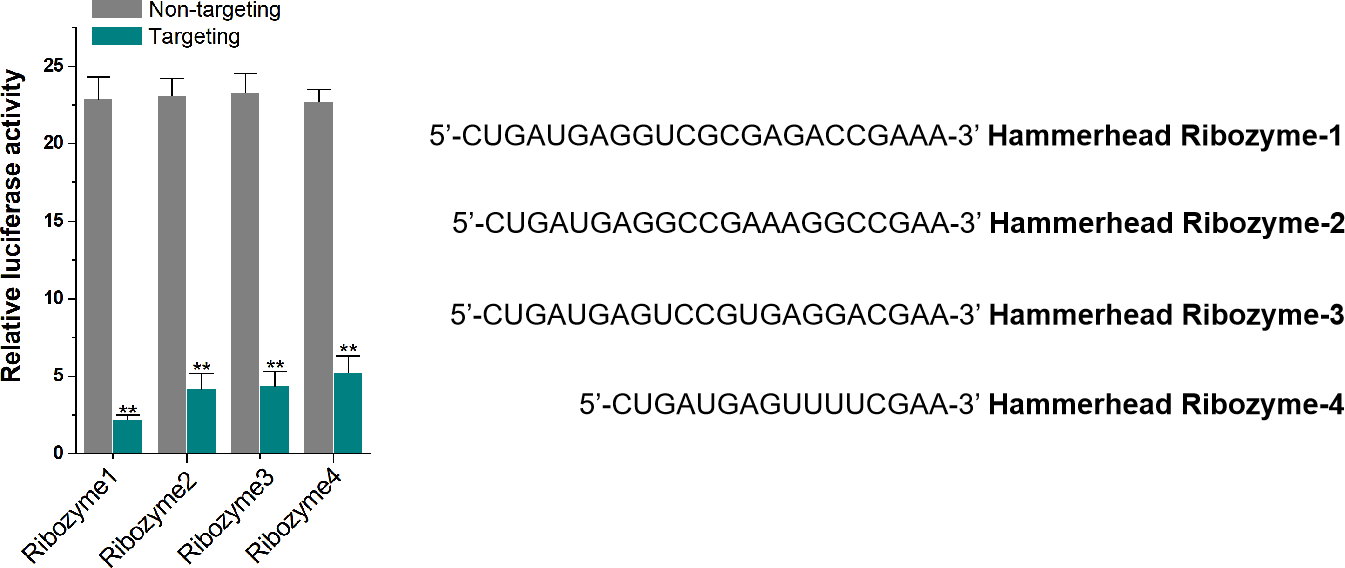


**Figure S4.** **Comparison of knockdown effects of different versions of hammerhead ribozymes in the CCRS system.** Knockdown of Rluc expression using CCRS with differnt versions of hammerhead ribozymes. The sequences of the used ribozymes were also listed. The relative expression level of Rluc was determined by luciferase activity assay. Fluc was used as the internal control. Each experiment was performed in triplicate for five independent times. Each error bar indicates the variation between the means of five independent experiments. **, p value < 0.01, relative to the control using a two-tailed t test.





**Figure S5. Targeting endogenous transcripts with CCRS in primary cultured cells.** Knockdown of six endogenous transcripts following transfection of fibroblasts was assayed using qRT-PCR. The results are shown as the mean ± SD. Each experiment was performed in triplicate five independent times. Each error bar indicates the variation between the means of five independent experiments. *, p value < 0.05, and **, p value < 0.01, relative to the CCRS group using a two-tailed t-test.


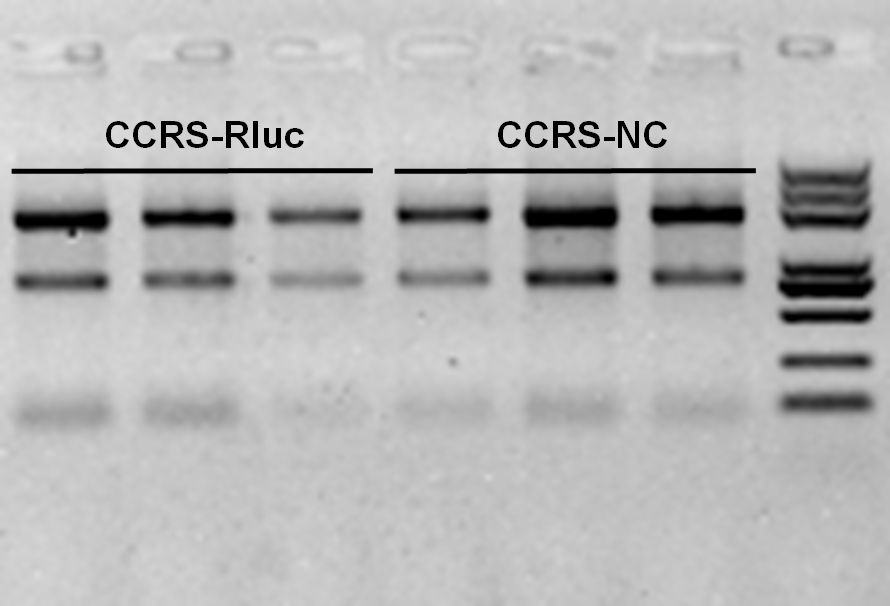


**Figure S6. RNA-denaturing gel electrophoresis examining RNA integrity.** Total RNA was collected and RNA-denaturing gel electrophoresis was applied to examine RNA integrity in HEK-293 cells transfected with targeting and nontargeting crRNAs 48 h post-transfection.

**
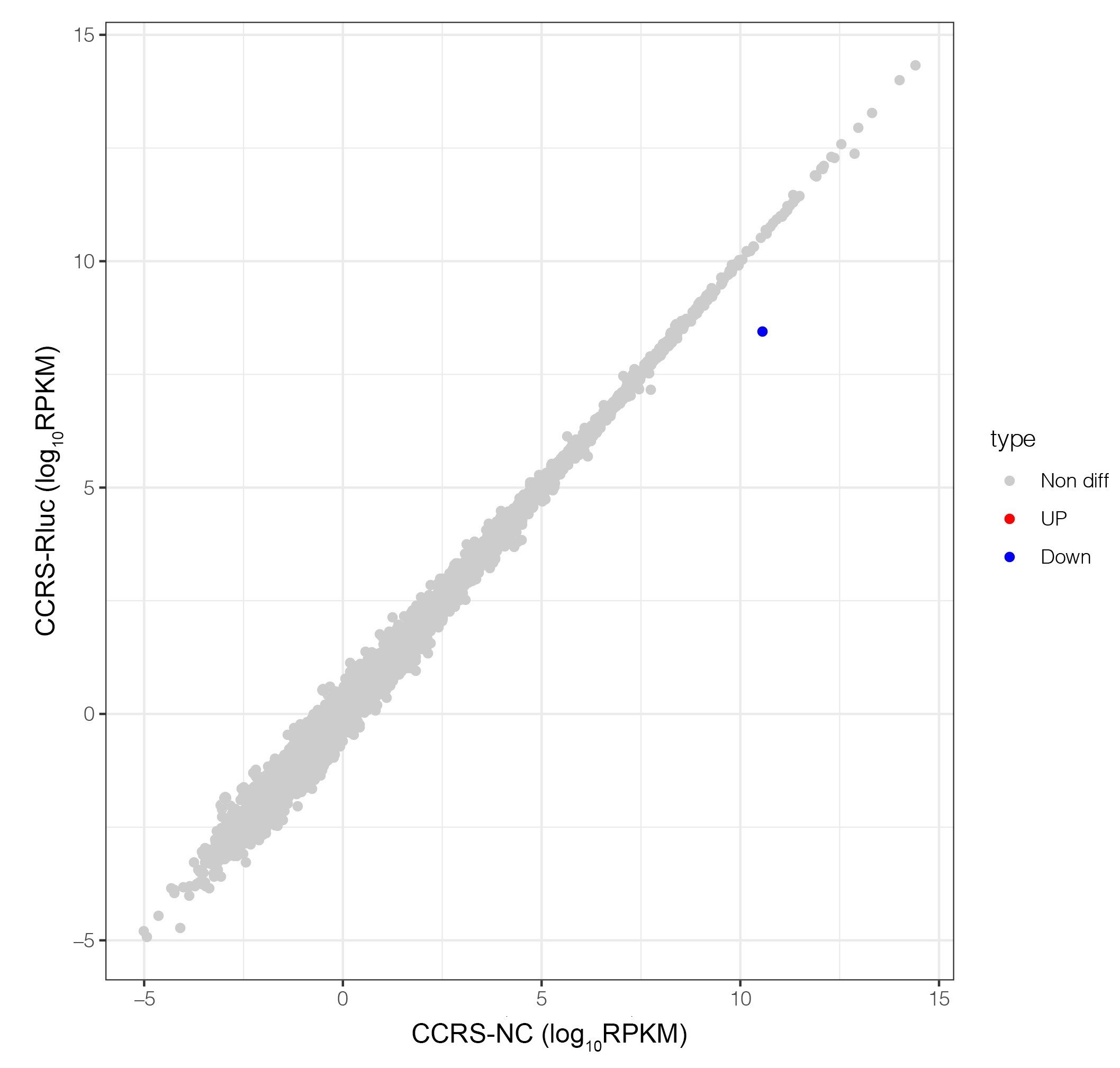
**

**Figure S7. Analysis of the specificity of CCRS-mediated knockdown.** HEK 293 cells expressing CasRx with the crRNA-ribozyme or the negative control were analyzed using RNA-Seq technology. According to the FPKRM values, only the expression of the Rluc (blue dot) was remarkably inhibited.

**
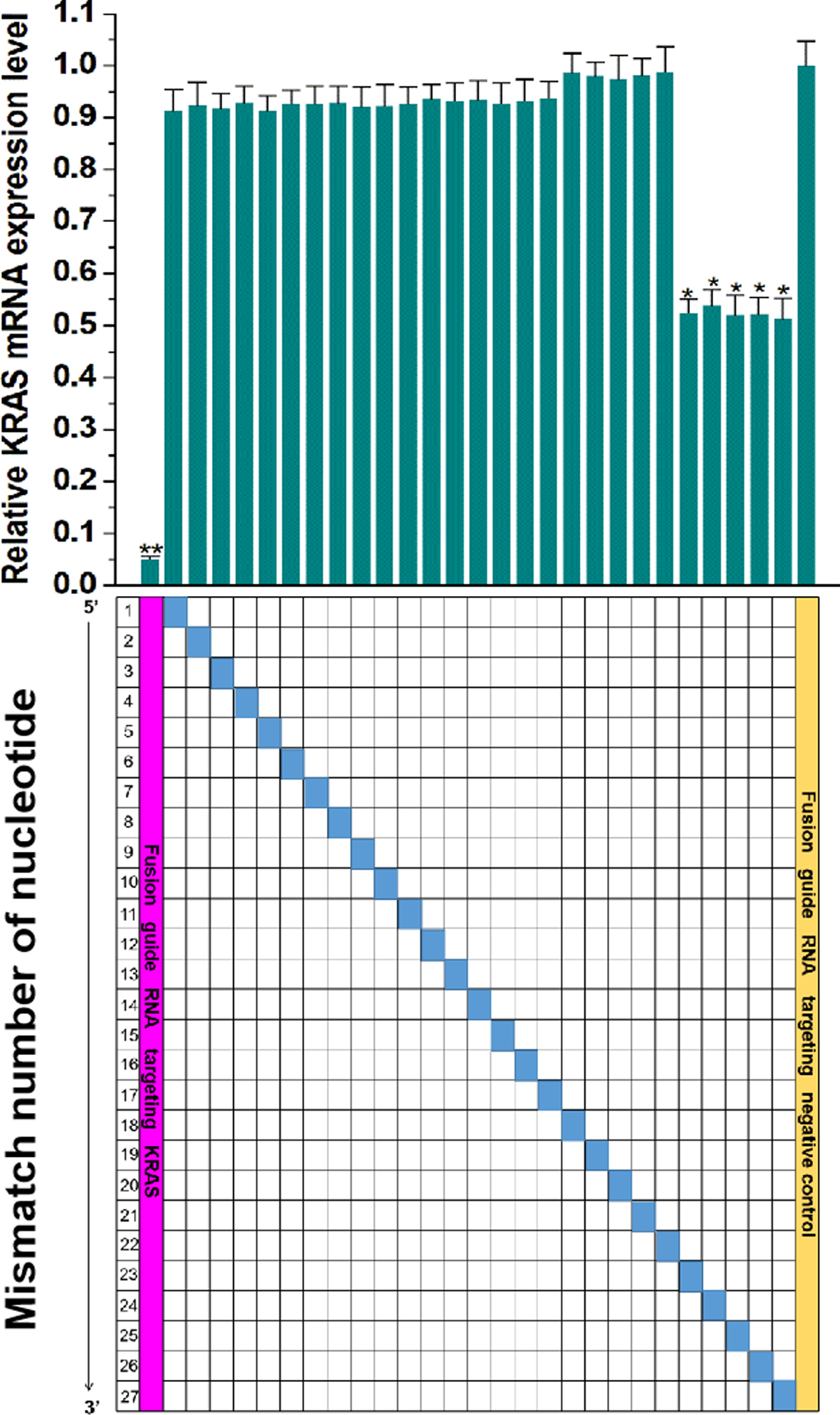
**

**Figure S8.** **Evaluation of CCRS knockdown specificity on endogenous gene.** Knockdown of KRAS mRNA evaluated with the fusion guides of CCRS containing single mismatches at varying positions across the antisense sequence. Data are expressed as the mean ± SD. Each experiment was performed in triplicate for five independent times. Each error bar indicates the variation between the means of five independent experiments. *, p value < 0.05, and **, p value < 0.01, relative to the control using a two-tailed t test.


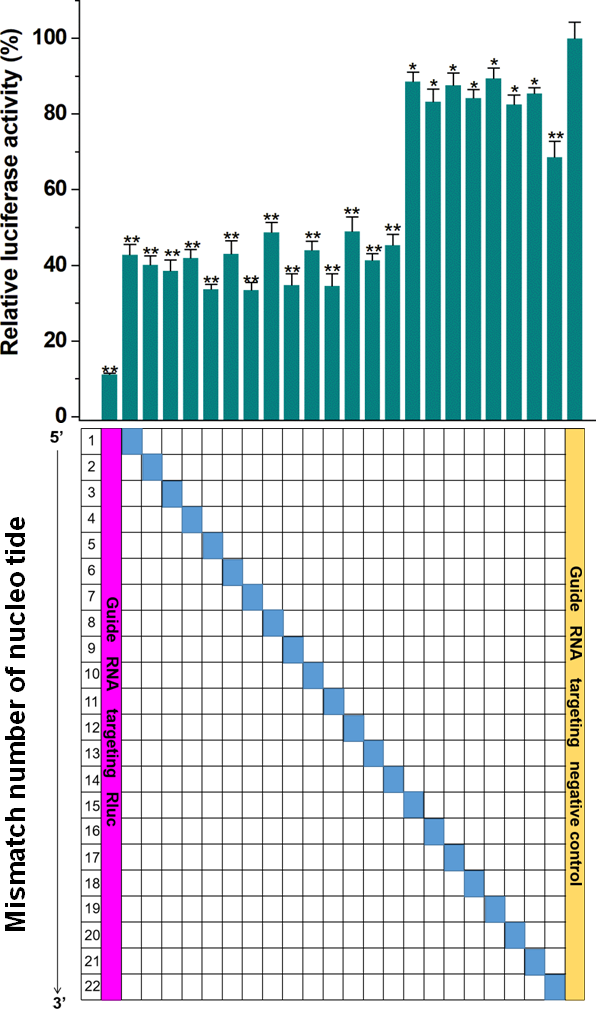


**Figure S9.** **Evaluation of CasRx knockdown specificity on luciferase gene.** Knockdown of Rluc mRNA evaluated with the guides of CasRx containing single mismatches at varying positions across the antisense sequence. Data are expressed as the mean ± SD. Each experiment was performed in triplicate for five independent times. Each error bar indicates the variation between the means of five independent experiments. *, p value < 0.05, and **, p value < 0.01, relative to the negative control using a two-tailed t test.





**Figure S10.** **qRT-PCR analysis of relative target RNA knockdown by CasRx and shRNA.** Rrepression efficiency values were the decline percentages (%) of transcript expression levels relative to the corresponding negative control. Data are expressed as the mean ± SD. Each experiment was performed in triplicate for five independent times. Each error bar indicates the variation between the means of five independent experiments.


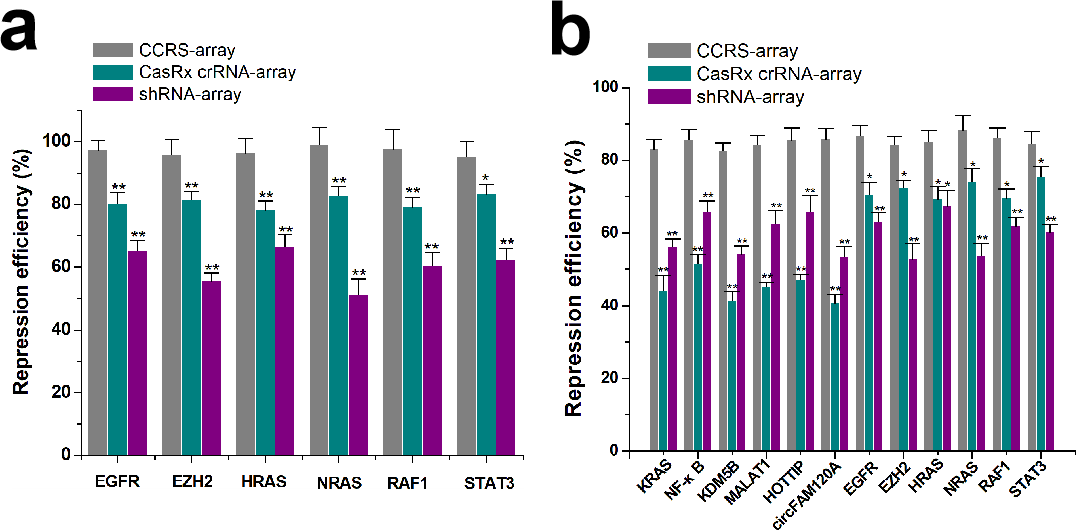


**Figure S11. Targeting other endogenous transcripts with CCRS. (a)** Knockdown of six endogenous transcripts following transfection of HEK293 was assayed using qRT-PCR. **(b)** Knockdown of twelve endogenous transcripts following transfection of HEK293 was assayed using qRT-PCR. The results are shown as the mean ± SD. Each experiment was performed in triplicate five independent times. Each error bar indicates the variation between the means of five independent experiments. *, p value < 0.05, and **, p value < 0.01, relative to the CCRS group using a two-tailed t-test.





**Figure S12.** **qRT-PCR analysis of relative target RNA knockdown by AAV-CCRS and other existing technologies.** Rrepression efficiency values were the decline percentages (%) of transcript expression levels relative to the corresponding negative control. Data are expressed as the mean ± SD. Each experiment was performed in triplicate for five independent times. Each error bar indicates the variation between the means of five independent experiments. *, p value < 0.05, and **, p value < 0.01, relative to the control using a two-tailed t test.


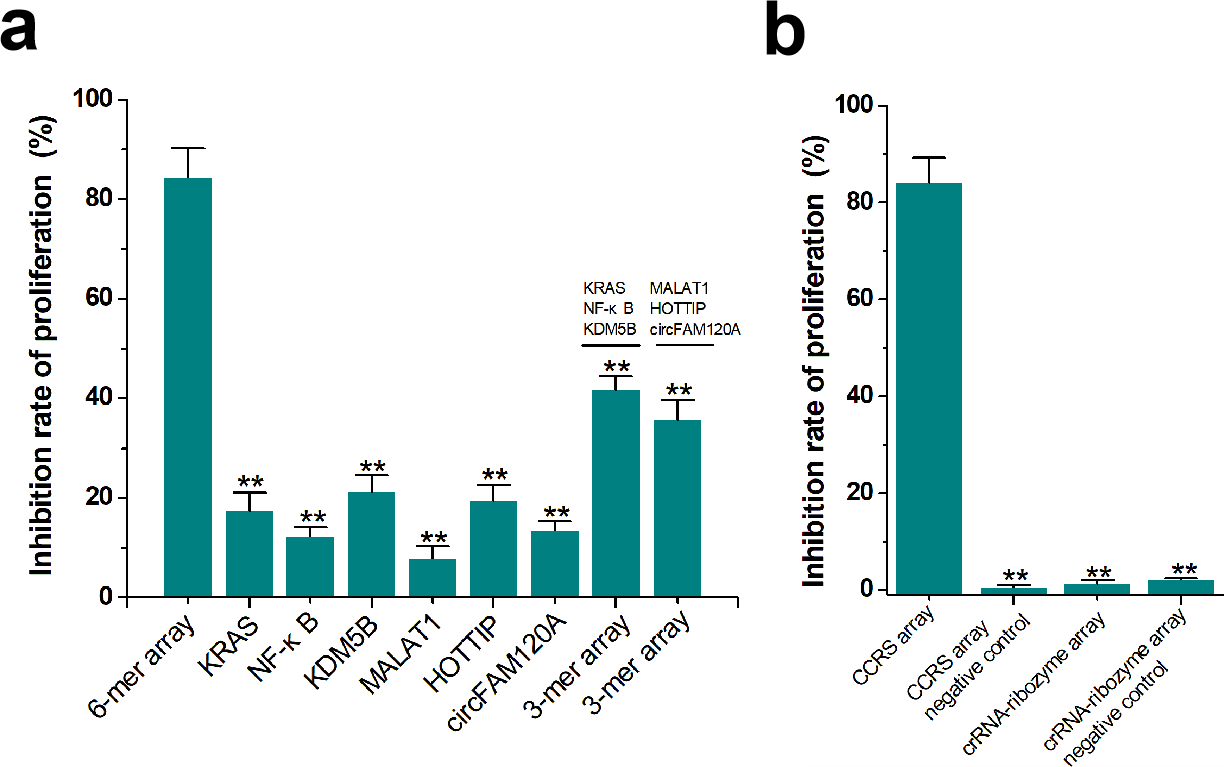


**Figure S13. The inhibition rate (%) of T24 cell proliferation. (a)** 6-mer arrays were compared with individual guides and smaller arrays. **(b)** Detection of possible cytotoxicity caused by ribozymes. Cell proliferation was determined using the CCK-8 assay, and the inhibition rate (%) at 72h after AAV transduction was then calculated. Data are expressed as the mean ± SD. Each experiment was performed in triplicate for five independent times. Each error bar indicates the variation between the means of five independent experiments. **, p value < 0.01, relative to the 6-mer array group using a two-tailed t test.

**

**

**Figure S14.** **ELISA assay on Caspase-3 activity in T24 cells treated by AAV-CCRS and other existing technologies.** Relative Caspase-3 activity is shown. Data are expressed as the mean ± SD. Each experiment was performed in triplicate for five independent times. Each error bar indicates the variation between the means of five independent experiments. *, p value < 0.05, and **, p value < 0.01, relative to the control using a two-tailed t test.


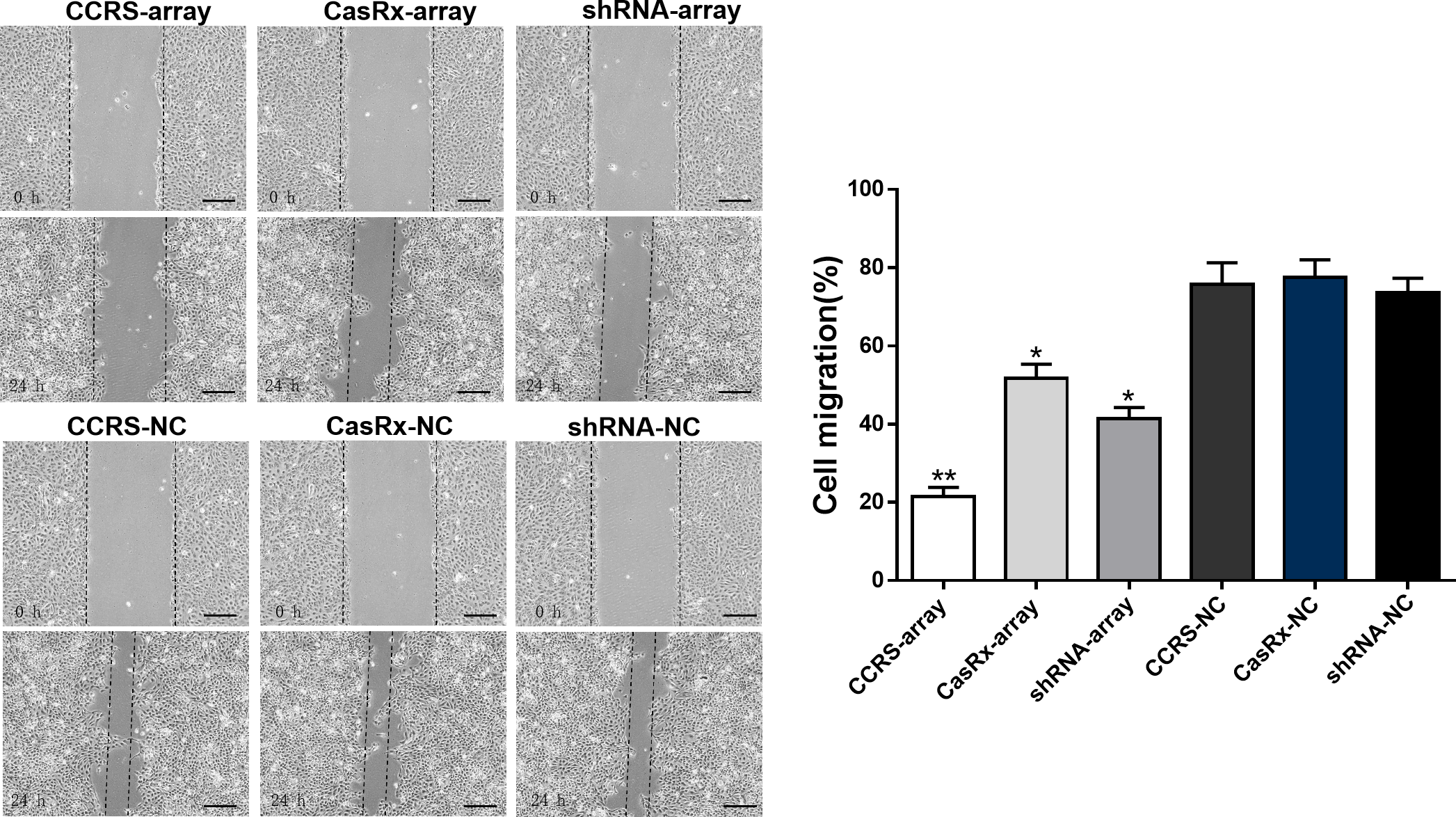


**Figure S15.** **AAV-CCRS efficiently inhibited T24 cell migration.** Migrations of the transduced T24 cells were measured using the scratch assay. Results were shown as the mean ± SD. Each experiment was performed for five independent times. Each error bar indicates the variation between the means of five independent experiments. *, p value < 0.05, and **, p value < 0.01, using a two-tailed *t* test.


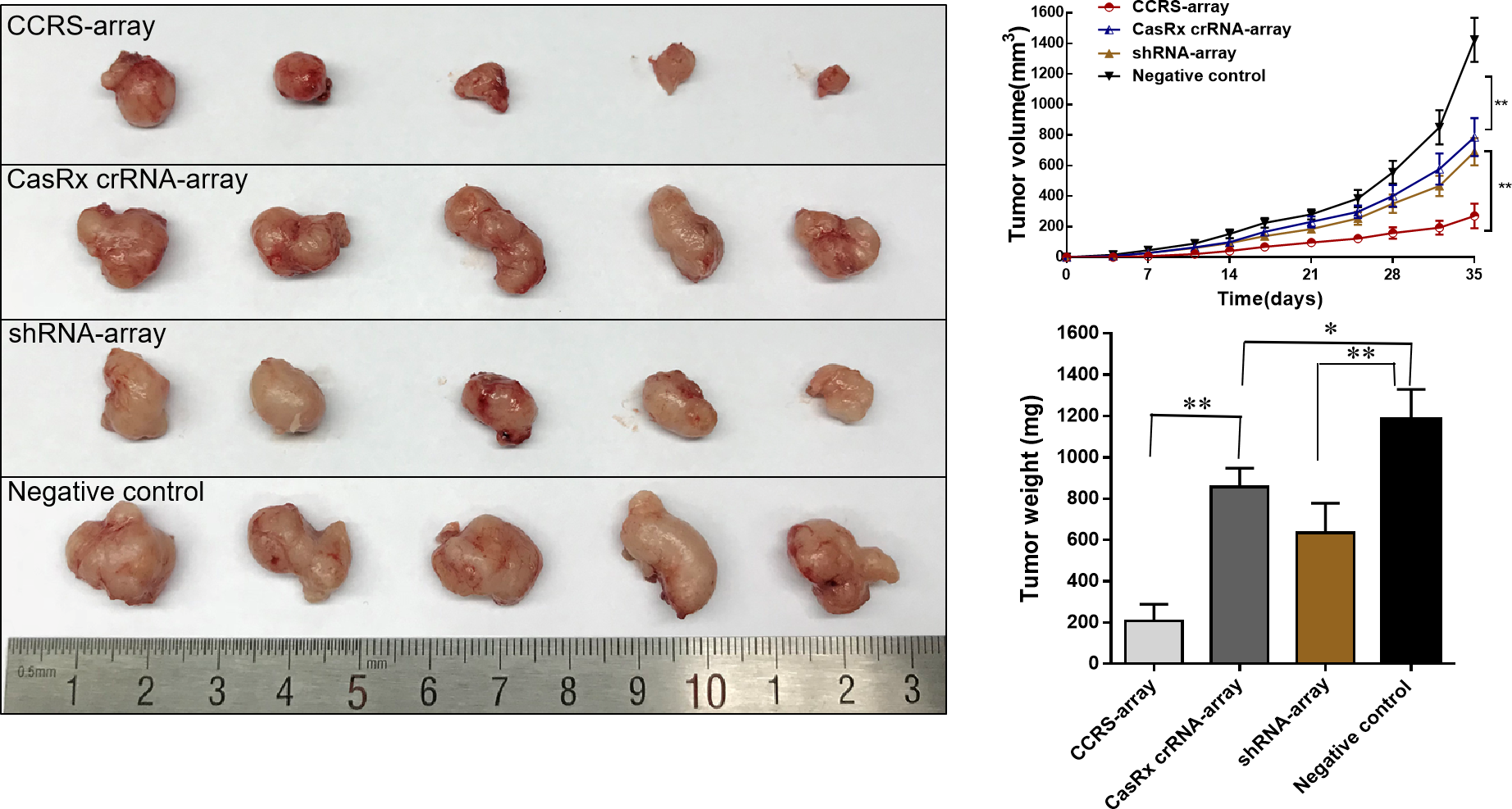


**Figure S16.** **AAV-CCRS efficiently inhibited *in vivo* tumor growth.** The tumor volume and weight were measured at the indicated time points after mice were transplanted. The *in vivo* growth of tumors treated with the AAV-CCRS was dramatically slower than those treated with other existing technologies and the negative control. Data are shown as the mean of mean ± SD. *p < 0.05 and **p < 0.01, between the groups, using a two-tailed t-test.


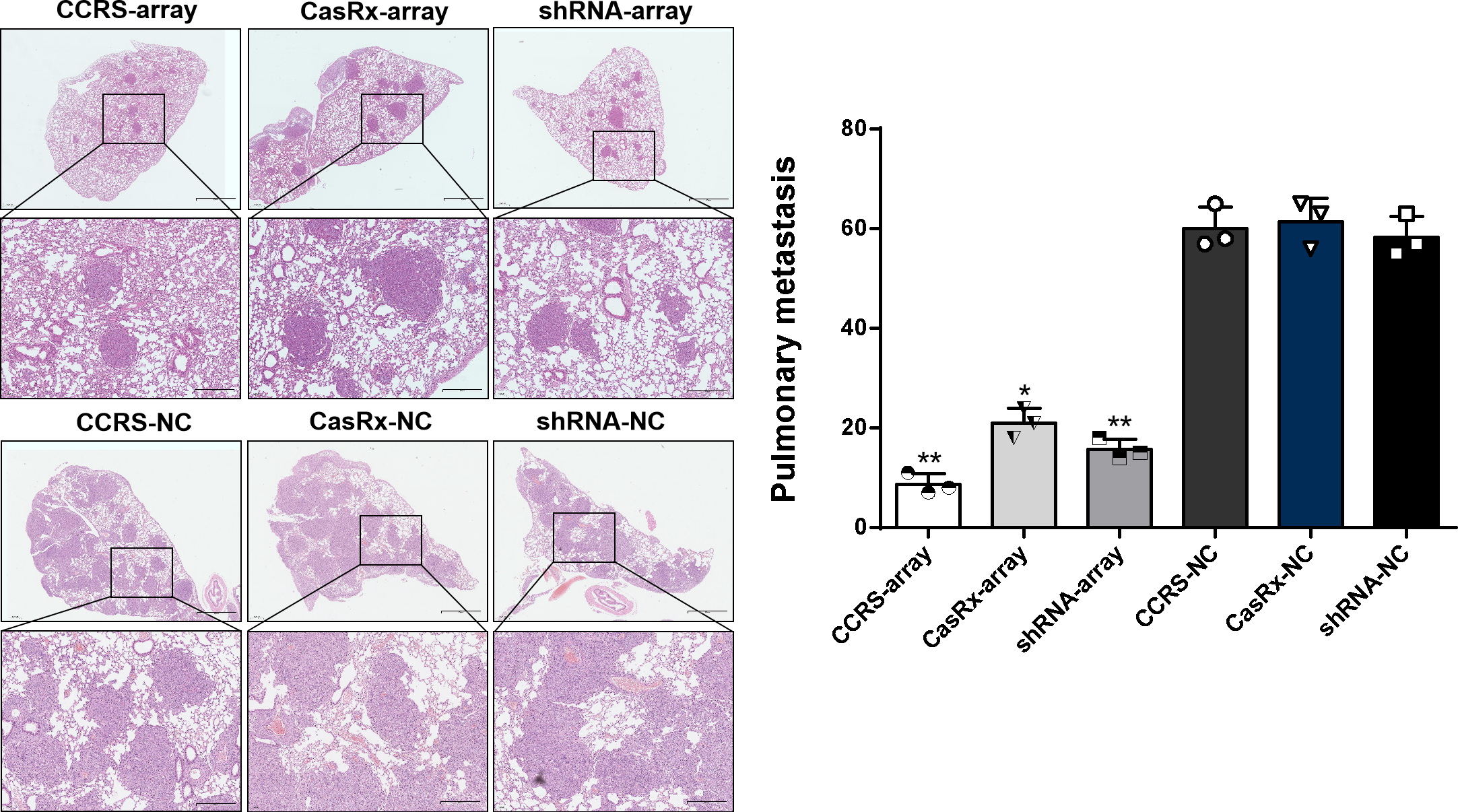


**Figure S17.** **Histopathological inspection of the mouse lungs treated with AAVs.** Lungs were examined with H & E staining and lung sections from mice injected with different AAVs were analyzed at 4 weeks after treatment. The pulmonary metastases with various sizes were observed. *, p value < 0.05, and **, p value < 0.01, using a two-tailed *t* test.
