# Supplementary material for: Enhanced RNA knockdown efficiency with engineered fusion guide RNAs that function with both CRISPR-CasRx and hammerhead ribozyme
Source: Genome Biol. 2023 Jan 17;24:9. doi: 10.1186/s13059-023-02852-w (PMC9843992; doi:10.1186/s13059-023-02852-w)
Supplement: Supplementary file 2 — Additional file 2: Table S1. The cDNA sequences of the engineered elements used in this study. [file 13059_2023_2852_MOESM2_ESM.doc]

**Table S1. The cDNA sequences of the engineered elements used in this study.**

| Names | Sequences |
| --- | --- |
| CCRS-Rluc | CACTAGTGCGAATTTGCACTAGTCTAAAACGCGTTTGCGTTGCTCGGGGTCGCTGATGAGGTCGCGAGACCGAAACACCT |
| CasRx crRNA-Rluc | CACTAGTGCGAATTTGCACTAGTCTAAAACGCGTTTGCGTTGCTCGGGGTCG |
| Antisense ribozyme-Rluc | GCGTTTGCGTTGCTCGGGGTCGCTGATGAGGTCGCGAGACCGAAACACCT |
| Mutated Antisense ribozyme-Rluc | GCGTTTGCGTTGCTCGGGGTCGTCAGCAGGGTCGCGAGACCAGGACACCT |
| shRNA-Rluc | GCGTTTGCGTTGCTCGGGGTCGTACACCT |
| CCRS-KRAS | CACTAGTGCGAATTTGCACTAGTCTAAAACGTATCGTCAAGGCACTCTTGCCCTGATGAGGTCGCGAGACCGAAACGCCA |
| CasRx crRNA-KRAS (22nt spacer) | CACTAGTGCGAATTTGCACTAGTCTAAAACGTATCGTCAAGGCACTCTTGCC |
| CasRx crRNA-KRAS (28nt spacer) | CACTAGTGCGAATTTGCACTAGTCTAAAACGTATCGTCAAGGCACTCTTGCCTACGCC |
| Antisense ribozyme-KRAS | GTATCGTCAAGGCACTCTTGCCCTGATGAGGTCGCGAGACCGAAACGCCA |
| shRNA-KRAS | GTATCGTCAAGGCACTCTTGCCTACGCCA |
| CCRS-NF-κB | CACTAGTGCGAATTTGCACTAGTCTAAAACAATACTTCTGGATTAAATATTGCTGATGAGGTCGCGAGACCGAAATGAGT |
| CasRx crRNA-NF-κB (22nt spacer) | CACTAGTGCGAATTTGCACTAGTCTAAAACAATACTTCTGGATTAAATATTG |
| CasRx crRNA-NF-κB (28nt spacer) | CACTAGTGCGAATTTGCACTAGTCTAAAACAATACTTCTGGATTAAATATTGTATGAG |
| Antisense ribozyme-NF-κB | AATACTTCTGGATTAAATATTGCTGATGAGGTCGCGAGACCGAAATGAGT |
| shRNA-NF-κB | AATACTTCTGGATTAAATATTGTATGAGT |
| CCRS-KDM5B | CACTAGTGCGAATTTGCACTAGTCTAAAACCGCCCAGCGGGCCCGGGCCCCCCTGATGAGGTCGCGAGACCGAAAGGGGC |
| CasRx crRNA-KDM5B (22nt spacer) | CACTAGTGCGAATTTGCACTAGTCTAAAACCGCCCAGCGGGCCCGGGCCCCC |
| CasRx crRNA-KDM5B (28nt spacer) | CACTAGTGCGAATTTGCACTAGTCTAAAACCGCCCAGCGGGCCCGGGCCCCCGAGGGG |
| Antisense ribozyme-KDM5B | CGCCCAGCGGGCCCGGGCCCCCCTGATGAGGTCGCGAGACCGAAAGGGGC |
| shRNA-KDM5B | CGCCCAGCGGGCCCGGGCCCCCGAGGGGC |
| CCRS-MALAT1 | CACTAGTGCGAATTTGCACTAGTCTAAAACGTTGCTAAAATGGCGCTGCGCTCTGATGAGGTCGCGAGACCGAAAAGAGG |
| CasRx crRNA-MALAT1 (22nt spacer) | CACTAGTGCGAATTTGCACTAGTCTAAAACGTTGCTAAAATGGCGCTGCGCT |
| CasRx crRNA-MALAT1 (28nt spacer) | CACTAGTGCGAATTTGCACTAGTCTAAAACGTTGCTAAAATGGCGCTGCGCTTAAGAG |
| Antisense ribozyme-MALAT1 | GTTGCTAAAATGGCGCTGCGCTCTGATGAGGTCGCGAGACCGAAAAGAGG |
| shRNA-MALAT1 | GTTGCTAAAATGGCGCTGCGCTTAAGAGG |
| CCRS-HOTTIP | CACTAGTGCGAATTTGCACTAGTCTAAAACTCCTGTGGTCTCACTGCAGAAACTGATGAGGTCGCGAGACCGAAAGCCGC |
| CasRx crRNA-HOTTIP (22nt spacer) | CACTAGTGCGAATTTGCACTAGTCTAAAACTCCTGTGGTCTCACTGCAGAAA |
| CasRx crRNA-HOTTIP (28nt spacer) | CACTAGTGCGAATTTGCACTAGTCTAAAACTCCTGTGGTCTCACTGCAGAAATAGCCG |
| Antisense ribozyme-HOTTIP | TCCTGTGGTCTCACTGCAGAAACTGATGAGGTCGCGAGACCGAAAGCCGC |
| shRNA-HOTTIP | TCCTGTGGTCTCACTGCAGAAATAGCCGC |
| CCRS-circFAM120A | CACTAGTGCGAATTTGCACTAGTCTAAAACGGTGATCCTCAATGCTCTGTGCCTGATGAGGTCGCGAGACCGAAACCTAA |
| CasRx crRNA-circFAM120A (22nt spacer) | CACTAGTGCGAATTTGCACTAGTCTAAAACGGTGATCCTCAATGCTCTGTGC |
| CasRx crRNA-circFAM120A (28nt spacer) | CACTAGTGCGAATTTGCACTAGTCTAAAACGGTGATCCTCAATGCTCTGTGCAACCTA |
| Antisense ribozyme-circFAM120A | GGTGATCCTCAATGCTCTGTGCCTGATGAGGTCGCGAGACCGAAACCTAA |
| shRNA-circFAM120A | GGTGATCCTCAATGCTCTGTGCAACCTAA |
| CCRS-EGFR | CACTAGTGCGAATTTGCACTAGTCTAAAACCGCTCACACCGTGCGGGGGGCGCTGATGAGGTCGCGAGACCGAAAGGCTG |
| CasRx crRNA-EGFR | CGCTCACACCGTGCGGGGGGCG |
| Antisense ribozyme-EGFR | CGCTCACACCGTGCGGGGGGCGCTGATGAGGTCGCGAGACCGAAAGGCTG |
| shRNA-EGFR | CGCTCACACCGTGCGGGGGGCGGAGGCTG |
| CCRS-EZH2 | CACTAGTGCGAATTTGCACTAGTCTAAAACGCCAACAAACTGGTCCCTTCTCCTGATGAGGTCGCGAGACCGAAAATTTC |
| CasRx crRNA-EZH2 | GCCAACAAACTGGTCCCTTCTC |
| Antisense ribozyme-EZH2 | GCCAACAAACTGGTCCCTTCTCCTGATGAGGTCGCGAGACCGAAAATTTC |
| shRNA-EZH2 | GCCAACAAACTGGTCCCTTCTCAGATTTC |
| CCRS-HRAS | CACTAGTGCGAATTTGCACTAGTCTAAAACCACCACCAGCTTATATTCCGTCCTGATGAGGTCGCGAGACCGAAACGCTC |
| CasRx crRNA-HRAS | CACCACCAGCTTATATTCCGTC |
| Antisense ribozyme-HRAS | CACCACCAGCTTATATTCCGTCCTGATGAGGTCGCGAGACCGAAACGCTC |
| shRNA-HRAS | CACCACCAGCTTATATTCCGTCATCGCTC |
| CCRS-NRAS | CACTAGTGCGAATTTGCACTAGTCTAAAACTTCATCTACAAAGTGGTTCTGGCTGATGAGGTCGCGAGACCGAAATAGCT |
| CasRx crRNA-NRAS | TTCATCTACAAAGTGGTTCTGG |
| Antisense ribozyme-NRAS | TTCATCTACAAAGTGGTTCTGGCTGATGAGGTCGCGAGACCGAAATAGCT |
| shRNA-NRAS | TTCATCTACAAAGTGGTTCTGGATTAGCT |
| CCRS-RAF1 | CACTAGTGCGAATTTGCACTAGTCTAAAACTGAGGGAGCGGGAGGCGGTCACCTGATGAGGTCGCGAGACCGAAATCGGC |
| CasRx crRNA-RAF1 | TGAGGGAGCGGGAGGCGGTCAC |
| Antisense ribozyme-RAF1 | TGAGGGAGCGGGAGGCGGTCAC  CTGATGAGGTCGCGAGACCGAAA  TCGGC |
| shRNA-RAF1 | TGAGGGAGCGGGAGGCGGTCACATTCGGC |
| CCRS-STAT3 | CACTAGTGCGAATTTGCACTAGTCTAAAACGTGTGTCAAGCTGCTGTAGCTGCTGATGAGGTCGCGAGACCGAAATCCAT |
| CasRx crRNA-STAT3 | GTGTGTCAAGCTGCTGTAGCTG |
| Antisense ribozyme-STAT3 | GTGTGTCAAGCTGCTGTAGCTGCTGATGAGGTCGCGAGACCGAAATCCAT |
| shRNA-STAT3 | GTGTGTCAAGCTGCTGTAGCTGATTCCAT |
