# Supplementary material for: Enhanced RNA knockdown efficiency with engineered fusion guide RNAs that function with both CRISPR-CasRx and hammerhead ribozyme
Source: Genome Biol. 2023 Jan 17;24:9. doi: 10.1186/s13059-023-02852-w (PMC9843992; doi:10.1186/s13059-023-02852-w)
Supplement: Supplementary file 3 — Additional file 3: Table S2. Primer sequences used in real-time quantitative PCR. [file 13059_2023_2852_MOESM3_ESM.doc]

**Table S2. Primer sequences used in real-time quantitative PCR.**

| Names | Sequences |
| --- | --- |
| KRAS-F | TGTGTCTCATATCAGGTTGACGA |
| KRAS-R | CAAGAGTCGAGTGTGGTCTCA |
| NF-κB-F | GTTGAGAGGTATATGGGATTAG |
| NF-κB-R | CACTCCAACCTTCTCACCAT |
| KDM5B-F | CCATAGCCGAGCAGACTGG |
| KDM5B-R | GGATACGTGGCGTAAAATGAAGT |
| MALAT1-F | CATTCGCTTAGTTGGTCTAC |
| MALAT1-R | TTCTACCGTTTTTAGCTTC |
| HOTTIP-F | GTGGGGCCCAGACCCGC |
| HOTTIP-R | AATGATAGGGACACATCGGGGAACT |
| circFAM120A-F | AGATCTGGCTTCCTTTCACTGGA |
| circFAM120A-R | CCGTTCCGGCTCAGTTTTAGG |
| EGFR-F | AGGCACGAGTAACAAGCTCAC |
| EGFR-R | ATGAGGACATAACCAGCCACC |
| EZH2-F | AATCAGAGTACATGCGACTGAGA |
| EZH2-R | GCTGTATCCTTCGCTGTTTCC |
| HRAS-F | ATGACGGAATATAAGCTGGTGGT |
| HRAS-R | GGCACGTCTCCCCATCAATG |
| NRAS-F | TGAGAGACCAATACATGAGGACA |
| NRAS-R | CCCTGTAGAGGTTAATATCCGCA |
| RAF1-F | GGGAGCTTGGAAGACGATCAG |
| RAF1-R | ACACGGATAGTGTTGCTTGTC |
| STAT3-F | CAGCAGCTTGACACACGGTA |
| STAT3-R | AAACACCAAAGTGGCATGTGA |
| Rluc crRNA-F | CACTAGTGCGAATTTGCACTAGTCT |
| Rluc crRNA-R | ACCCCGAGCAACGCAAA |
| Rluc crRNA-probe | CACTAGTCTAAAACGC |
| Rluc crRNA-ribozyme-F | GCGAATTTGCACTAGTCTAAAACG |
| Rluc crRNA-ribozyme-R | AGGTGTTTCGGTCTCGCG |
| Rluc crRNA-ribozyme-probe | CTCATCAGCGACCC |
| GAPDH-F | CGCTCTCTGCTCCTCCTGTTC |
| GAPDH-R | ATCCGTTGACTCCGACCTTCAC |
