# Supplementary material for: Enhanced RNA knockdown efficiency with engineered fusion guide RNAs that function with both CRISPR-CasRx and hammerhead ribozyme
Source: Genome Biol. 2023 Jan 17;24:9. doi: 10.1186/s13059-023-02852-w (PMC9843992; doi:10.1186/s13059-023-02852-w)
Supplement: Supplementary file 4 — Additional file 4. Review history. [file 13059_2023_2852_MOESM4_ESM.docx]

Review History

**First round of review**

**Reviewer 1**

**Are you able to assess all statistics in the manuscript, including the appropriateness of statistical tests used?** There are no statistics in the manuscript.

**Comments to author:**

Enhanced RNA knockdown efficiency with engineered fusion guide RNAs that function
with both CRISPR-CasRx and hammerhead ribozyme

I read the manuscript by Zhan et al., with great interest in which authors claim to enhance the RNA knockdown efficiency of the already known CRISPR/CasRx system by engineering its crRNA. The authors have shown successfully that the engineered crRNA can guide CasRx and Ribozyme simultaneously, thus improving a specific transcript's knockdown. With multiplexed targeting (6-mer), they have observed even an improved efficiency compared to single transcript targeting.
Following in vitro observations, the authors further expanded their system to in vivo and targeted multiple oncogenic genes to open up new ways for cancer gene therapy. A decrease in the tumour growth suggested that the CCRS system could efficiently knock down multiple genes simultaneously. The authors have successfully executed all the experiments, and the manuscript may be accepted for publication in Genome Biology after addressing the following comments.

1. The authors observe high knockdown efficiency in a multiplex system compared to targeting a single transcript. Also, the knockdown efficiency of CCRS against a single target is slightly better than CasRx alone. Authors may comment on this.

2. The authors mention the dilution effect of CasRx in multiplex targeting. They explain that the observed lower activity of CasRx than shRNA is because CasRx activity is diluted, i.e. processing of crRNA and targeting. But then again, the same rule applies to the CCRS system as well. Authors may explain why CCRS system efficiency remains similar in multiplex targeting compared to single targeting.

3. CasRx has the ability to process poly crRNAs. The engineering combined RNA may also be prone to processing activity by CasRx. Did the authors keep that in mind, and what measures they have taken to protect it from the CasRx processing activity? It is crucial to show the crRNAs on the gel in the absence and presence of CasRx compared to crRNA alone (i.e. regular crRNA of CasRx) to conclude that the crRNA remains intact in vivo.

**Reviewer 2**

**Are you able to assess all statistics in the manuscript, including the appropriateness of statistical tests used?** Yes, and I have assessed the statistics in my report.

**Comments to author:**

In this manuscript by Zhan et al., described the development of an updated RNA editing system through combining the RNA guided Cas13 nuclease with antisense ribozyme (called CCRS) to achieve higher efficiency than solo Cas13 or ribozymes. The authors further showed that CCRS were effective to treat tumor growth through targeting oncogenic genes in a bladder cancer xenograft mouse model. The authors also showed that the CCRS tool had advantages for efficient targeting of long non-coding RNAs or multiple transcripts simultaneously. It is an interesting study, but the CCRS is only a prototype, extensive optimization, comparation with other tools and evaluation of off-targeting effects should be provided.

Major points:

1. Although the authors presented important data to show combination of CasRx with ribozyme had higher efficiency than solo CasRx or ribozyme, there is no direct evidence to show the mechanism of CCRS system. Does the ribozyme sequence fusion with crRNA still has a ribozyme activity? In vitro evidence should be provided, otherwise the increase of activity could be the modification of crRNA, since modifications of the 3' or 5' sequence of sgRNAs to increase the RNA stability showed increased activity. Moreover, how is the in vitro activity of CCRS? Does the binding of CasRx affected ribozyme activity? In vitro study of the CCRS system will provide evidence whether both of the enzymes digested the target mRNA or they function independently. Additionally, it is also necessary to test whether CCRS still has higher activity than other solo systems when point mutations to kill the ribozyme activity were introduction into hammerhead ribozyme.

2. As the CCRS system combined to strategy for targeted RNA digestion, whether independently transfect the CasRx/crRNA and ribozyme could achieve higher inhibition? It seems that CCRS is conceptually more simple than independent CasRx/ribozyme system, but the target sequences of CasRx and ribozyme in the CCRS are stringent associated (the localization of the digestion sites of each system are located relatively fixed). As we all know the efficiency is variant at different target site both for CasRx and ribozyme, if CasRx and ribozyme are separate sequences, we can screen for highly efficient crRNA and ribozyme target independently and combined the most efficient ones. However, for the CCRS system, if we choose a highly efficient crRNA target, it will be difficult to find a highly efficient site of ribozyme in adjacent sequence.

3. The authors emphasized the high efficiency of their CCRS system. However, they only tested knockdown of the same six transcript in vitro as well as in vivo. For rigorous comparison between CasRx and CCRS knockdown efficiency, the CCRS should have been investigated in more endogenous mRNAs. Moreover, in published reports, the activity of CasRx was much higher compared to the data in this study. Several reported target sites should be employed to compare with CCRS.

4. Why choose hammerhead ribozyme for the study? How about other ribozymes? Even for hammerhead ribozyme, it also has different versions or sequences, which one is the best? More optimization studies should be provided to educate the reader getting easily adapted the system.


5. Off-target effects of CasRx were tested, but how about the specificity of the crRNA-ribozyme? To further investigate the specificity of CCRS, transcriptome-wide mRNA sequencing is recommended to comprehensively evaluate off-target effects of CCRS. Without this information, the description " CCRS has a low off-target potential." was not supported properly.

6. I totally disagree with the sentence in the abstract, as "Among all members of Cas13, CasRx has the highest efficiency and specificity in targeting RNAs", since many Cas13 variants, such as Cas13bt, Cas13x and Cas13y, have been shown to be very efficient. The introduction and discussion of Cas13 family should cite the new variants. As Cas13bt, Cas13x and Cas13y are compact Cas13 members, they have advantages compared to CasRx. Could these compact Cas13 variants also be adapted to CCRS system?

7. The introduction is not well organized, almost no introduction for ribozyme. In line 56-58, reference 8 is not a study to support "ribozymes with cleavage activity have been designed to be fused with antisense RNA". The cited study has demonstrated the application of external guide sequences (EGSs) variants to efficiently direct human RNase P to cleave a target mRNA encoding human cytomegalovirus (HCMV) in vitro. It is not a study to increase ribozyme activity.

8. In line 64, "RNA interference (RNAi) is the highly efficient and specific degradation…" and In line 70, "However, this technology may have large off-target effects and certain cytotoxicity". It is confused RNAi is highly specific or has large off-target effects?

**Authors Response**

**Point-by-point responses to the reviewers’ comments:**

*Reviewer #1: Enhanced RNA knockdown efficiency with engineered fusion guide RNAs that function
with both CRISPR-CasRx and hammerhead ribozyme

I read the manuscript by Zhan et al., with great interest in which authors claim to enhance the RNA knockdown efficiency of the already known CRISPR/CasRx system by engineering its crRNA. The authors have shown successfully that the engineered crRNA can guide CasRx and Ribozyme simultaneously, thus improving a specific transcript's knockdown. With multiplexed targeting (6-mer), they have observed even an improved efficiency compared to single transcript targeting.
Following in vitro observations, the authors further expanded their system to in vivo and targeted multiple oncogenic genes to open up new ways for cancer gene therapy. A decrease in the tumour growth suggested that the CCRS system could efficiently knock down multiple genes simultaneously. The authors have successfully executed all the experiments, and the manuscript may be accepted for publication in Genome Biology after addressing the following comments.

1.The authors observe high knockdown efficiency in a multiplex system compared to targeting a single transcript. Also, the knockdown efficiency of CCRS against a single target is slightly better than CasRx alone. Authors may comment on this.*

**Response: Thanks very much for your important questions. Indeed, in our results, we observed that the knockdown of CCRS was better than that of ribozyme or CasRx alone. Especially in the multiplex system, CCRS performs particularly well. In our design, CCRS uses a combination of two RNA degradation systems, CasRx and hammerhead ribozyme, to target RNA. So its knockdown effect is better than that of the wild-type CasRx. In order to further confirm our point of view, in this revision, we also introduced point mutations into the binding sequence of the core sequence of hammerhead ribozyme in CCRS, and found that the knockdown efficiency of CCRS was significantly weakened (Supplementary Fig.3). By using the constructed mutants as controls, we confirmed that CCRS indeed utilizes ribozymes and CasRx simultaneously to achieve high-efficiency knockdown of target genes. Even in the multiplex system, CCRS can still preserve high-efficiency knockdown although CasRx is diluted to a certain extent. It may be noted that the knockdown effect of CCRS in the multiplex system is basically the same as that of CCRS targeting a single target. The CasRx used in the multiplex system was fused with the NLS signal, so the knockdown effect was a little higher than that of the CCRS without NLS.**

*2.The authors mention the dilution effect of CasRx in multiplex targeting. They explain that the observed lower activity of CasRx than shRNA is because CasRx activity is diluted, i.e. processing of crRNA and targeting. But then again, the same rule applies to the CCRS system as well. Authors may explain why CCRS system efficiency remains similar in multiplex targeting compared to single targeting.*

**Response: Thanks very much for your important questions. Although CasRx can be used to simultaneously target multiple transcripts, its knockdown efficiency decreases with an increasing number of guides targeting different transcripts. This phenomenon may be related to the dilution effect, because the CasRx protein not only processes the precursor crRNA, but also binds and cleaves a variety of different transcripts. Our results show that the performance of CCRS in this case is more effective than that of wild -type CasRx, which may be attributed to the ribozyme activity and the longer complementary antisense sequence of CCRS that enhances the binding of CasRx to target RNA. However, the performance of CasRx was only slightly improved when we used crRNA with the same length as the antisense sequence of CCRS, suggesting that the ribozyme and antisense sequences may synergistically promote the binding of RNA targets. We discuss this in the Discussion section of the manuscript.
Of course, if the number of targets is further increased, the efficiency of CCRS may still decline. In this revision, we constructed two crRNA arrays independently driven by the U6 promoter in the CCRS system, each targeting 6 different genes. It was found that the knockdown effect of CCRS was reduced when the number of targets reached 12, but the effect of CCRS was still higher than that of wild-type CasRx (Supplementary Fig.11b).**


*3.CasRx has the ability to process poly crRNAs. The engineering combined RNA may also be prone to processing activity by CasRx. Did the authors keep that in mind, and what measures they have taken to protect it from the CasRx processing activity? It is crucial to show the crRNAs on the gel in the absence and presence of CasRx compared to crRNA alone (i.e. regular crRNA of CasRx) to conclude that the crRNA remains intact in vivo.*

**Response: Thank you very much for your constructive professional advice on the potential cleavage activity of the CasRx protein on the crRNA of the CCRS system. The mature crRNA is divided into a constant direct repeat (DR) region and a spacer region complementary to the RNA target. Specific recognition and binding of the constant 5′ DR region within their cognate crRNAs is a key requirement for all class 2 CRISPR effectors. In the Cas13d binary structure, there are multiple residues that interact in either a base- or backbone-specific manner with the DR of crRNA. The RNA secondary structure formed by these specific sequences ensures that the CasRx protein cleaves only at the 5' end of the DR structure (Cell. 2018;175(1):212-223.e17. doi:10.1016/j.cell.2018.09.001).
In order to further clarify whether the crRNA in CCRS may be degraded by CasRx, we designed probes and amplification primers according to the sequences of wild-type crRNA and crRNA-ribozyme in CCRS, respectively, and detected them by fluorescence quantitative PCR. The results indicated that transfection of CasRx expression plasmid didn’t have an observable effect on the expression levels of crRNA-ribozyme and wild-type crRNA (Supplementary Fig.2a). Also, the biochemical cleavage reaction revealed that the crRNA-ribozyme remained intact in the presence of CasRx (Supplementary Figure 2b).**


*Reviewer #2: In this manuscript by Zhan et al., described the development of an updated RNA editing system through combining the RNA guided Cas13 nuclease with antisense ribozyme (called CCRS) to achieve higher efficiency than solo Cas13 or ribozymes. The authors further showed that CCRS were effective to treat tumor growth through targeting oncogenic genes in a bladder cancer xenograft mouse model. The authors also showed that the CCRS tool had advantages for efficient targeting of long non-coding RNAs or multiple transcripts simultaneously. It is an interesting study, but the CCRS is only a prototype, extensive optimization, comparation with other tools and evaluation of off-targeting effects should be provided.

Major points:

1.Although the authors presented important data to show combination of CasRx with ribozyme had higher efficiency than solo CasRx or ribozyme, there is no direct evidence to show the mechanism of CCRS system. Does the ribozyme sequence fusion with crRNA still has a ribozyme activity? In vitro evidence should be provided, otherwise the increase of activity could be the modification of crRNA, since modifications of the 3' or 5' sequence of sgRNAs to increase the RNA stability showed increased activity. Moreover, how is the in vitro activity of CCRS? Does the binding of CasRx affected ribozyme activity? In vitro study of the CCRS system will provide evidence whether both of the enzymes digested the target mRNA or they function independently. Additionally, it is also necessary to test whether CCRS still has higher activity than other solo systems when point mutations to kill the ribozyme activity were introduction into hammerhead ribozyme.*

**Response: Thank you very much for your valuable suggestions for further validation of the cleavage mechanism of CCRS. Based on literature reports, we introduced key mutaions into the hammerhead ribozymes. A dead version of CasRx that cannot cleave RNA was also used as a control. We found that the knockdown efficiency of CCRS was significantly affected by using either the dead version of the ribozyme or dCasRx. We also found that the knockdown efficiency of CCRS was close to that of wild-type CasRx when the ribozyme was mutated. Interestingly, the knockdown efficiency of CCRS was higher than that of the wild-type antisense ribozyme even when CasRx was mutated. Without the addition of dCasRx, the knockdown efficiency of crRNA-ribozyme was comparable to that of the wild-type ribozyme. These results suggest that CCRS should utilize CasRx and ribozyme simultaneously to cleave the targets (Supplementary Fig.3).**

*2. As the CCRS system combined to strategy for targeted RNA digestion, whether independently transfect the CasRx/crRNA and ribozyme could achieve higher inhibition? It seems that CCRS is conceptually more simple than independent CasRx/ribozyme system, but the target sequences of CasRx and ribozyme in the CCRS are stringent associated (the localization of the digestion sites of each system are located relatively fixed). As we all know the efficiency is variant at different target site both for CasRx and ribozyme, if CasRx and ribozyme are separate sequences, we can screen for highly efficient crRNA and ribozyme target independently and combined the most efficient ones. However, for the CCRS system, if we choose a highly efficient crRNA target, it will be difficult to find a highly efficient site of ribozyme in adjacent sequence.*

**Response: Thank you very much for another constructive suggestion on the efficiency of CCRS. During this revision, we tried to compare the knockdown efficiency of the CCRS group with the CasRx crRNA+ribozyme group (CasRx crRNA and ribozyme on the same vector are independently driven by separate U6 promoters). We found that the knockdown efficiency of CCRS was more efficient than that of the CasRx+ribozyme group (Supplementary Fig.3). As we mentioned in our response to the previous question, the knockdown efficiency of CCRS was also better than that of the antisense ribozyme group when dCasRx was co-transfected (Supplementary Fig.3). Of course, further study on the optimal selection of CCRS targets is still needed. We stated this in the Discussion section: "Although our results suggested that CCRS is conceptually simpler than the independent CasRx/ribozyme system to design, the target sequences of CasRx and ribozyme in the CCRS are stringently associated and may be selected carefully. In order to optimize the effectiveness of the CCRS system, further research is needed to determine how to select the best targets."**

*3.The authors emphasized the high efficiency of their CCRS system. However, they only tested knockdown of the same six transcript in vitro as well as in vivo. For rigorous comparison between CasRx and CCRS knockdown efficiency, the CCRS should have been investigated in more endogenous mRNAs. Moreover, in published reports, the activity of CasRx was much higher compared to the data in this study. Several reported target sites should be employed to compare with CCRS.*

**Response: Thank you very much for your valuable suggestions, and we do agree that more targets need to be added to further verify the knockdown efficiency of the CCRS system. According to previous reports, we added EGFR, EZH2, HRAS, NRAS, RAF1, and STAT3 as new intervention targets for CCRS, and used CasRx and shRNA as controls. The results showed that the average knockdown efficiencies of CCRS (>95%), CasRx (about 80%) and shRNA (ranging from 51%-67%) decreased sequentially (Supplementary Fig.11a).**

*4.Why choose hammerhead ribozyme for the study? How about other ribozymes? Even for hammerhead ribozyme, it also has different versions or sequences, which one is the best? More optimization studies should be provided to educate the reader getting easily adapted the system.*

**Response: Thanks very much for your important questions and valuable advices. The hammerhead ribozyme is the most intensively studied and applied ribozyme to date. This ribozyme is the smallest of the naturally occurring ribozymes, and the development of hammerhead ribozyme-based technology that cleave target RNA molecules in trans was a major advancement that made possible the use of ribozyme technology for practical applications. Therefore, we chose hammerhead ribozyme for this study. We did compare the efficiency of many different versions of hammerhead ribozymes. During this revision, we also included these results. You can see that in the CCRS system, fusion of different versions of hammerhead ribozymes into the crRNA of the CasRx system can reduce the expression of target genes to varying degrees, and the version we showed in the original manuscript has the highest knockdown efficiency (Supplementary Fig.4). So we had used this ribozyme for subsequent cytology and animal experiments.**

*5.Off-target effects of CasRx were tested, but how about the specificity of the crRNA-ribozyme? To further investigate the specificity of CCRS, transcriptome-wide mRNA sequencing is recommended to comprehensively evaluate off-target effects of CCRS. Without this information, the description " CCRS has a low off-target potential." was not supported properly.*

**Response: Thank you very much for your important professional advice on further validation of the specificity of CCRS. HEK 293 cells expressing CasRx with the crRNA-ribozyme or the negative control were analyzed using RNA-Seq technology. According to the FPKRM values, only the expression of the Rluc was remarkably inhibited, demonstrating the specificity of CCRS system (Supplementary Fig.7). We have also revised the description in the manuscript: "these data show that CCRS is very sensitive to mismatches in general and may have a low off-target potential, consistent with previous studies of CasRx [22,23]."**

*6.I totally disagree with the sentence in the abstract, as "Among all members of Cas13, CasRx has the highest efficiency and specificity in targeting RNAs", since many Cas13 variants, such as Cas13bt, Cas13x and Cas13y, have been shown to be very efficient. The introduction and discussion of Cas13 family should cite the new variants. As Cas13bt, Cas13x and Cas13y are compact Cas13 members, they have advantages compared to CasRx. Could these compact Cas13 variants also be adapted to CCRS system?*

**Response: Thank you very much for pointing out the problems with the description about the efficiency of CasRx. In addition to CasRx, there are many variants of Cas13, which are indeed very competitive. The mentioned sentence in the abstract has been modified to "Among all members of Cas13, CasRx degrades RNA in human cells with high precision and effectiveness". We have also made corrections to the description regarding the efficiency of CasRx in other relevant places in the manuscript. Also, we have added descriptions of Cas13bt, Cas13x and Cas13y in the Introduction and Discussion sections, and cited the latest references. Specifically in the Discussion section we mentioned: "The antisense ribozyme can also be applied to guide RNAs of other Cas13 enzymes besides CasRx. Many Cas13 variants, such as Cas13bt, Cas13x and Cas13y, have been shown to be very efficient. As Cas13bt , Cas13x and Cas13y are compact Cas13 members, they may have advantages compared to CasRx [16, 17, 41, 42]. Indeed, if new nucleases of the Cas13 family with higher RNA knockdown efficiency are found, the CCRS strategy will have better application prospects".**

*7.The introduction is not well organized, almost no introduction for ribozyme. In line 56-58, reference 8 is not a study to support "ribozymes with cleavage activity have been designed to be fused with antisense RNA". The cited study has demonstrated the application of external guide sequences (EGSs) variants to efficiently direct human RNase P to cleave a target mRNA encoding human cytomegalovirus (HCMV) in vitro. It is not a study to increase ribozyme activity.*

**Response: Thank you very much for pointing out the organizational problems in the introduction section of the manuscript. We have added a description of ribozymes as you suggested and added a corresponding reference: Fedor MJ, Williamson JR. The catalytic diversity of RNAs. Nat Rev Mol Cell Biol. 2005 May;6(5):399-412 . We also realized that there was some misunderstanding of the main findings of the original cited reference 8, so we replaced it with a new reference: "Abounader R, Montgomery R, Dietz H, Laterra J. Design and expression of chimeric U1/ribozyme transgenes. Methods Mol Biol. 2004;252:209-19”. The revised manuscript states: "Natural or in vitro-evolved ribozymes are RNA molecules that possess the ability to catalyze specific biochemical reactions. Their most common activities include the cleavage or ligation of RNA and DNA, and the formation of peptide bonds [8]. In order to further enhance the inhibitory effects of antisense RNAs, self-cleaving hammerhead ribozymes with fast cleavage kinetics have been designed to be fused with antisense RNA [9]...".**

*8.In line 64, "RNA interference (RNAi) is the highly efficient and specific degradation…" and In line 70, "However, this technology may have large off-target effects and certain cytotoxicity". It is confused RNAi is highly specific or has large off-target effects?*

**Response: Thank you very much for pointing out the inconsistency here. This is an error caused by our negligence in writing the manuscript. We have removed the word "specific" from the introduction to RNAi. The revised sentence is "RNA interference (RNAi) is the highly efficient degradation of homologous mRNA induced by double-stranded small interfering RNA (siRNA)".**

**Second round of review**

**Reviewer 2**

The authors have addressed most of my concerns, and no more comments.
